# Lignocellulosic Biomass-Derived Carbon Electrodes for Flexible Supercapacitors: An Overview

**DOI:** 10.3390/ma14164571

**Published:** 2021-08-14

**Authors:** Wenxin Hu, Ruifang Xiang, Jiaxian Lin, Yu Cheng, Chunhong Lu

**Affiliations:** 1 Key Laboratory of Textile Science & Technology, Donghua University, Ministry of Education, Shanghai 201620, China; 2180004@mail.dhu.edu.cn (W.H.); 2190025@mail.dhu.edu.cn (R.X.); 2190041@mail.dhu.edu.cn (J.L.); 2190053@mail.dhu.edu.cn (Y.C.); 2 College of Textiles, Donghua University, Shanghai 201620, China

**Keywords:** lignocellulose-derived carbon, electrode, flexible/wearable supercapacitor, electrochemical performance

## Abstract

With the increasing demand for high-performance electronic devices in smart textiles, various types of flexible/wearable electronic device (i.e., supercapacitors, batteries, fuel cells, etc.) have emerged regularly. As one of the most promising wearable devices, flexible supercapacitors from a variety of electrode materials have been developed. In particular, carbon materials from lignocellulosic biomass precursor have the characteristics of low cost, natural abundance, high specific surface area, excellent electrochemical stability, etc. Moreover, their chemical structures usually contain a large number of heteroatomic groups, which greatly contribute to the capacitive performance of the corresponding flexible supercapacitors. This review summarizes the working mechanism, configuration of flexible electrodes, conversion of lignocellulosic biomass-derived carbon electrodes, and their corresponding electrochemical properties in flexible/wearable supercapacitors. Technology challenges and future research trends will also be provided.

## 1. Introduction

With the rapid development of information, the demand for flexible/wearable electronic devices is increasing drastically. As a representative of wearable electronic devices by flexible textile manufacturing technology, smart textiles have drawn intense research interest. Smart textiles are defined as textile goods (i.e., woven, knitted or non-woven structures from fibers, filaments or yarns) that can interact with the environment/user [1]. The combination of conventional textiles and flexible wearable electronic devices (i.e., energy storage devices, sensors, etc.) is capable of accomplishing a spectrum of functions in various fields, such as health monitoring, medical care, sports, military, etc.

In this context, numerous efforts have been made in developing flexible and efficient energy storage systems (i.e., supercapacitors (SCs), batteries and fuel cells, etc.) for smart textiles. A good example is the supercapacitor, which is the main component of electrochemical (EC) energy storage devices. A supercapacitor, also known as ultracapacitor or electrochemical capacitor, has the characteristics of high power density, fast charge/discharge cycles, and good cycle performance (>100,000 cycles) [2,3,4,5]. Considerable research and development has focused on transforming a rigid capacitor into a flexible capacitor in recent years since the latter is lightweight, small and portable [6,7,8], and suitable for application in smart textiles. Two-dimensional (2D) planar structured flexible supercapacitors have been developed to be employed in 2D woven fabrics [9,10]. Three-dimensional (3D) carbon aerogels have also been applied in flexible SCs. To further integrate flexible SCs into fabric structure, it is of great importance to develop one-dimensional (1D) fiber-shaped supercapacitors (FSSCs) with good mechanical strength for textile manufacturing (i.e., weaving, knitting, braiding, etc.), and excellent EC performance for energy storage. Although studies regrading FSSCs for flexible electronic applications are proliferating, the development of wearable fiber-shaped or yarn-shaped SCs is still at an initial stage [11,12,13,14,15].

Apart from the flexible supercapacitor’s configuration, it is vital to achieve good EC performance for smart textiles application. Generally, EC performance of supercapacitors is attributed to the electrochemical stability, specific surface area (SSA) and electrical conductivity of electrode materials. To obtain flexible supercapacitors with high capacity and superior rate performance, more attention has been paid to the development of electrode materials with tunable composition and microstructure [16]. Among different electrode materials (i.e., carbon nanotubes (CNTs), activated carbon (AC), metal oxides, carbon fibers, conducting polymers, etc.), carbon materials, typically used in electrical double-layer capacitors (EDLCs), are prospective electrode materials for industrialization [2]. Carbon-based electrodes for supercapacitors typically possess these features: high SSA up to the order of 1000 m^2^/g, good conductivity and good electrolyte accessibility. However, the relatively high cost and elaborate preparation process of carbon materials hinder the wide application of flexible supercapacitors. Thus, there is significant motivation for investigations of carbon electrodes based on renewable resources and green, facile fabrication processes.

Lignocellulosic biomass-derived carbon can be potentially used in flexible supercapacitors due to their low cost, natural abundance and sustainability. The structures of lignocellulose stocks are typically composed of carbon (C), hydrogen (H) and heteroatoms of oxygen (O) [17], nitrogen (N), sulfur (S) and phosphorus (P), etc. In general, these heteroatoms can result in self-doped biomass-derived carbon after pyrolysis [18,19]. They often boost the electrical conductivity, improve the surface wettability, introduce pseudocapacitance, accelerate the charge transfer, and facilitate the electrode/electrolyte interface reactions in lignocellulose-derived carbon, thereby enhancing the EC performance of lignocellulosic biomass-derived carbon electrode-based supercapacitors [20,21,22]. The hierarchically intermeshed lignocellulose structures are held by covalent or non-covalent bonding. After carbonization/activation, lignocellulosic carbons with hierarchical pores (micro-, meso- and macropores) offer high SSA to accommodate ions, abundant channels for ions transportation and sufficient reservoir spaces for ion-buffering. Therefore, lignocellulosic carbons with interconnected hierarchical porous frameworks and high SSA can efficiently improve the EC performances of supercapacitors [23]. Thus, it is of great importance to review the current development of lignocellulose-derived carbon electrodes in flexible supercapacitors.

**Figure 1:**
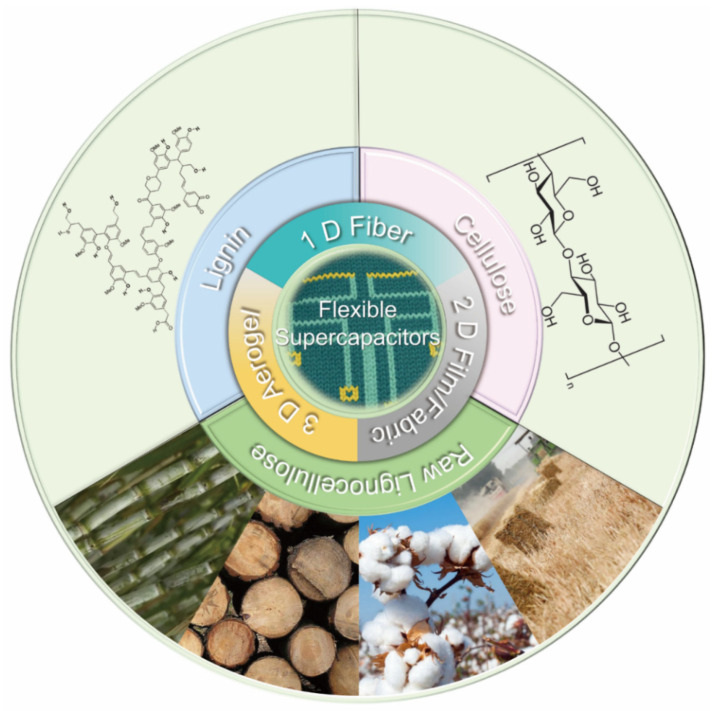
Overview diagram of lignocellulosic biomass-derived carbon electrodes for flexible supercapacitors (reproduced with permission from ref. [31] Copyright 2014 John Wiley and Sons) in this review.

Several published review articles have summarized various aspects of biomass-derived porous carbon including biomass species [17,24], fabrication methods [25,26], carbon architecture [27], influencing factors of EC performance (i.e., pore characteristics, heteroatom-doping, morphology, graphitization degree, etc.) [28], and wide applications in fields such as power generation and energy storage (i.e., lithium-metal or lithium-sulfur batteries, electrolyte reservoir, separator, supercapacitors, etc.) [29,30]. However, incomplete information is available in the current literature that comprehensively summarizes lignocellulosic biomass-derived carbon electrodes for flexible supercapacitors and their corresponding EC performance. Thus, the feasibility of using cellulose, lignin or raw lignocellulose stock as the major component to fabricate low-cost bio-based flexible supercapacitors (Figure 1) will be stressed. The novelty of this article review is that the relationship of the structure/functionality of lignocellulosic biomass precursor, lignocellulosic biomass-derived carbon/graphite electrode, and EC performance of the resulting flexible supercapacitors will be emphasized. In this review, an overview of the type and working mechanism of supercapacitors, and the configuration of flexible supercapacitors will first be introduced. Moreover, conversion of lignocellulose to carbon/graphite electrode and corresponding EC performance of the assembled flexible supercapacitors will be discussed. Lastly, the review concludes with future perspectives on fabricating lignocellulose-derived carbon electrode for flexible supercapacitors.

## 2. Types and Working Mechanisms of Supercapacitors

**Figure 2:**
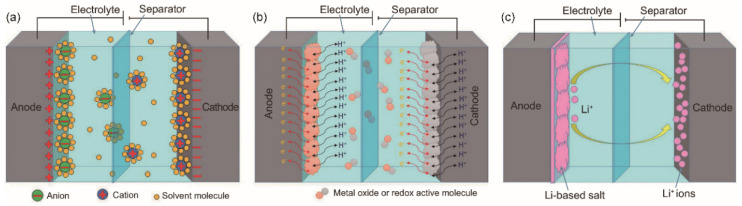
Energy storage principles of (**a**) electrical double-layer capacitors (EDLCs), (**b**) pseudocapacitors, and (**c**) asymmetric supercapacitors. Reprinted from ref. [32], an open-access article.

In general, there are three types of supercapacitor: EDLCs, pseudocapacitor and asymmetric supercapacitor [32]. The energy storage mechanism of EDLC (Figure 2a) is mainly based on the electrical double-layer theory, which involves the nanoscale charge separation, and the formation of electrochemical interface between an electrode and an electrolyte [33]. Since the physical charge transfer mechanism of EDLCs is non-faradaic and involves no chemical oxidation-reduction (redox) reaction, the life cycle of EDLCs is relatively long. Pseudocapacitors, also known as Faraday capacitors, involve faradaic redox reactions that mainly occur on the surface of the electrode materials (Figure 2b). The chemical reactions contribute to the pseudocapacitor’s higher energy density but lower life cycles in comparison with those of EDLCs [34]. The asymmetric supercapacitor (Figure 2c) is a combination of EDLC and pseudocapacitor. Its working mechanism is that the electrolyte ions are absorbed by one electrode in the capacitor, forming an electrical double-layer structure for energy storage. The pseudocapacitive electrode materials (i.e., metal oxides, conductive polymers or metal-doped carbons) involve an electrochemical reaction for energy storage. Thus, asymmetric supercapacitors possess the advantages of both EDLCs and pseudocapacitors due to different energy storage mechanisms of the positive and negative electrodes [35]. Compared with the EDLCs, asymmetric supercapacitors have higher energy density, higher working voltage, and the superior cycle stability.

## 3. Configuration of Flexible Electrodes in Supercapacitors

Compared with traditional rigid supercapacitors, flexible/wearable supercapacitors have shorter charge/discharge time, longer cycle life and other merits such as smaller size, lighter weight, and better flexibility [36,37,38,39]. The assembly of wearable supercapacitors typically involves three configurations, including 1D linear (fiber-shaped), 2D film-based planar and 3D aerogel interconnected porous structure. In this section, the configuration of flexible supercapacitors potentially for smart textiles applications based on 1D, 2D and 3D electrodes will be introduced.

### 3.1. Flexible One-Dimensional Fiber-Shaped Electrodes

One-dimensional (1D) fiber-shaped electrodes have good flexibility, small size, light weight, fast charge/discharge process, long service life and good stability [40,41]. More importantly, they can maintain stable EC performance during folding, stretching, compression and other deformation processes [42,43], thus showing broad application prospects in smart textiles.

**Figure 3:**
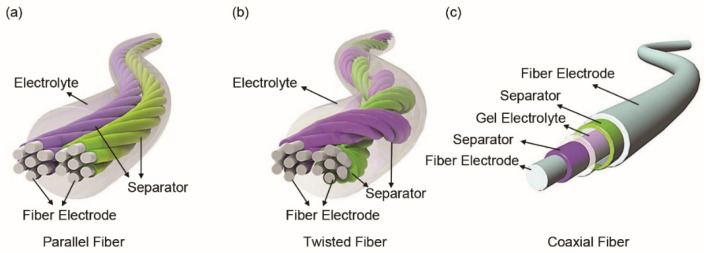
Assembly of fiber-shaped supercapacitors (FSSCs) with (**a**) parallel, (**b**) twisted and (**c**) coaxial structure. Reproduced with permission from ref. [45] Copyright 2019 John Wiley and Sons.

Structurally, FSSCs can be assembled from fiber electrodes in parallel, twisted or coaxial form [44]. The parallel structure is obtained by placing two fiber electrodes in parallel and encapsulating them with a solid electrolyte (Figure 3a). The assembled FSSCs can be used together in series or in parallel to meet specific energy and power requirements of microelectronics. The FSSCs with twisted structure are fabricated by winding fiber electrodes together (Figure 3b). This process is quite similar to the twisting of yarns in textile manufacturing process. This twisted structure, with a high direct contact area between the fiber electrodes, is beneficial for the electrochemical reaction during the charge/discharge process. A coaxial structure (Figure 3c), comprising of a core fiber electrode, a separator or solid-state electrolyte, and an outer electrode layer, is achieved by the layer-by-layer method. Compared with the aforementioned two structures, this coaxial structure has a much larger effective electrode contact area, which is more conducive to the adsorption/desorption of charges on the electrode surface and the redox reaction. Moreover, the assembled device is more stable under deformation since it has good integrity as a whole.

**Figure 4:**
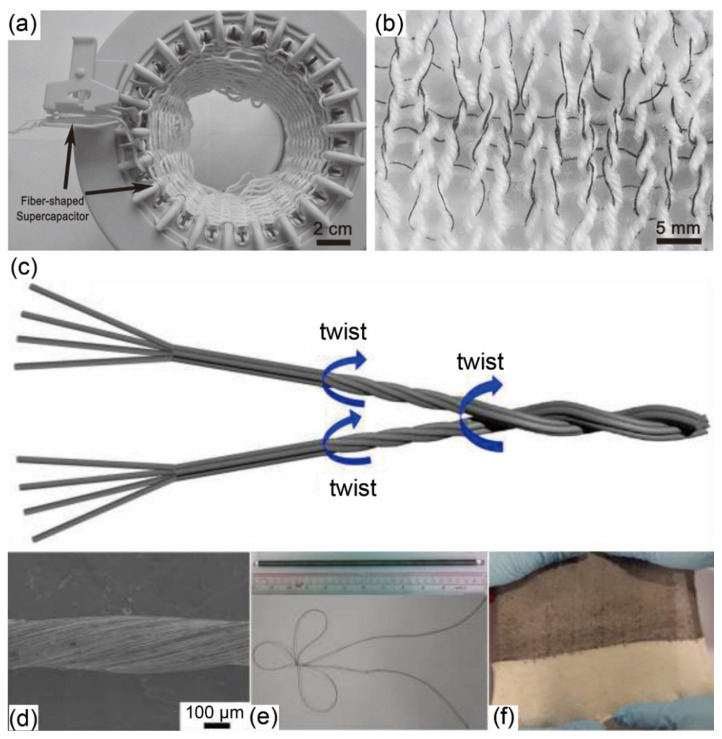
(**a**) Picture of the knitting process. (**b**) Cotton yarn (white) with integrated fiber-shaped supercapacitors (black). (**c**) Schematics of yarn fabrication. (**d**) Scanning electron microscopy (SEM) image of an as-drawn yarn. (**e**) A long yarn rolled on a rod and knotted. (**f**) A cloth knitted by the as-drawn yarn and cotton yarn. (**a**,**b**) Reproduced with permission from ref. [46] Copyright 2015 John Wiley and Sons. (**c**–**f**) Reproduced with permission from ref. [47] Copyright 2015 American Chemical Society.

One-dimensional (1D) FSSCs can be easily converted into energy storage yarns, which can be further integrated with traditional clothing [42,43] or fabricated into energy storage 2D fabrics (Figure 4a,b) [46] for smart textiles. For instance, Huang et al. [47] first loaded graphene onto cotton thread by the hydrothermal method, then grew manganese oxide (MnO_2_) and polypyrrole (PPy) in sequence through electrodeposition, and finally assembled the linear yarn electrodes (Figure 4c–e) to achieve supercapacitors that were knitted into smart textiles (Figure 4f). Lucas et al. [48] produced an all-in-one graphene/MnO_2_ composite FSSCs device. The core graphene fiber (GF) electrode was sequentially coated with a hierarchically nanostructured MnO_2_ layer, gel electrolyte of poly (vinyl alcohol)/phosphoric acid (PVA/H_3_PO_4_), and sheath GF electrode. The novel coaxial structure equipped the device with a high volumetric capacitance of 29.6 F/cm^3^ at a scan rate of 2 mV/s, and remarkable capacitance retention of 93% after 1000 cycles, making it potentially woven into smart textiles.

### 3.2. Flexible Two-Dimensional Planar Electrodes

Flexible supercapacitors potentially used in smart textiles can be directly obtained from 2D planar electrodes, which form a sandwich structure with a solid electrolyte in between. In general, 2D planar electrode, either in film or fabric form, is the most commonly used configuration for flexible supercapacitors.

Quite a few works in the literature deposited electroactive substances onto flexible current collectors to prepare 2D thin-film electrodes for flexible supercapacitors [49,50]. For instance, flexible supercapacitors were constructed by the intercalation of PVA/H_3_PO_4_ gel electrolyte between two drop-casted reduced graphene oxide (rGO) thin-film electrodes on a tin-doped indium oxide (In_2_O_3_)-coated polyethylene terephthalate (PET) flexible substrate [51]. However, when the wearable devices were subjected to repeated body deformation, the delamination between the 2D electrode and current collector may have occurred. This can adversely affect the comfort of smart textiles and the energy storage performance of the device. To prevent this delamination issue, one strategy is to develop a self-supporting or binder-free flexible 2D thin-film electrode. This method can reduce the volume occupied by the current collector, increase the utilization rate of the active material, and finally increase the specific capacity of the electrode [52]. For instance, transition metal oxide (TMO) such as vanadium pentoxide (V_2_O_5_) films were achieved via a thermal evaporation technique [53]. The 2D film-based symmetric device (V_2_O_5_/electrolyte/V_2_O_5_) had a thickness of ~540 nm, and its pseudocapacitive behavior contributed to a maximum areal specific capacitance of 9.7 mF/cm^2^ at a scan rate of 10 mV/s. The promising performance remained even after being bent at 60° and 120°, indicating great potential in smart textile applications. Furthermore, the delamination between self-supporting flexible 2D film electrode and separator can be prevented by the formation of good interfacial adhesion between the two. A good example is that the wearable supercapacitor was constructed by utilizing 2D lignosulfonate/single-wall carbon nanotube/holey reduced graphene oxide (Lig/SWCNT/HrGO) film as the electrode/current collector and high-strength cellulose hydrogel as the separator. The strong interaction between lignosulfonate and cellulose effectively resulted in smaller contact resistance between different layers and prevent the delamination between the electrode and the separator [54].

Two-dimensional (2D) film is also achieved by electrospun polymeric nanofibers, which form a membrane or non-woven mat. Electrospun polymeric nanofibers can be further carbonized to yield freestanding, binder-free and current collector-free carbon electrodes with porous structure and tunable porosity [55]. These non-woven electrodes offer shorter diffusion distance, and effectively decrease charge transfer resistance at their interface with the electrolyte to ensure superior rate capability [55]. In particular, lignocellulose is widely employed as the precursor of electrospun carbon nanofiber electrode of flexible supercapacitors, which will be introduced in detail in Section 5 of this article.

**Figure 5:**
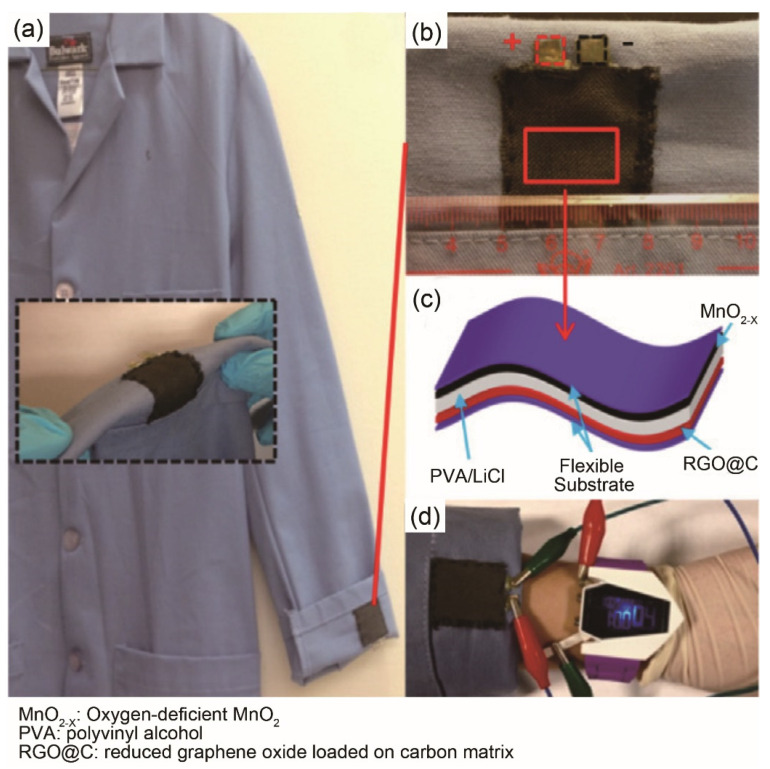
(**a**–**d**) The assembled wearable supercapacitor provides high energy/power capacity as worn in the real cloth. Reproduced with permission from ref. [56] Copyright 2014 Elsevier.

To successfully promote the application of flexible supercapacitor in smart textiles, it is necessary to achieve 2D fabric-based electrodes with conventional textile manufacturing techniques such as weaving, knitting or non-woven processing. Direct coating of existing fabric with electroactive material is a simple and facile approach. For example, Zhai et al. [56] used electrodeposition and heat treatment methods to obtain MnO_2_-deposited carbon cloth as a positive electrode, and graphene-coated carbon cloth as a negative electrode. The assembled asymmetric fabric-based wearable supercapacitors were achieved by soaking two electrodes and a separator in electrolyte solution (Figure 5). Norawish et al. [57] successfully fabricated a conductive cotton fabric electrode by screen-printing an ink containing conductive silver (Ag) and one of the electroactive materials-AC, graphene, and CNTs, onto the uncoated cotton fabric. Fabric-based symmetric supercapacitors were assembled by inserting a separator of uncoated cotton fabric containing an electrolyte into two coated conductive cotton cloths. It was found that the AC-based conductive cotton electrode displayed the highest areal specific capacitance of ~3290 mF/cm^2^ at a scan rate of 5 mV/s, as well as excellent long-term cyclic stability. However, the wash durability of the smart textiles from the coated fabric-based supercapacitors remains to be further explored.

Moreover, a fabric-based electrode can be obtained from flexible bio-based fabrics through a simple carbonization process, which is a cost-effective and sustainable route. Wang et al. [58] carbonized weft-knitted Modal fabric, and assembled inherently stretchable and conductive fabric-based supercapacitors with PVA/H_3_PO_4_ gel as an electrolyte/separator. This fabric-based supercapacitor exhibited a maximum specific capacitance of 7.5 mF/cm^2^ (1.2 F/g) at the scan rate of 10 mV/s, and a capacitance retention of 44% as the scan rate increased from 50 mV/s to 500 mV/s.

### 3.3. Flexible Three-Dimensional Aerogel Electrodes

To obtain 3D interconnected aerogel electrodes, two routes are typically used. One route is that carbon-based framework filled with a high concentration of solvents (i.e., water) is directly converted into aerogel by different drying methods (i.e., freeze-drying, supercritical drying) to retain the 3D hierarchically porous structure [55,59]. Another strategy is to construct a 3D crosslinked structure of precursor materials before the carbonization process [60]. Both strategies result in highly porous structure. In general, hierarchical pores containing micro- (<2 nm), meso- (2–50 nm) and macropores (>50 nm) are preferred. Macro-/mesopores facilitate the fast ion transport by serving as ion buffering reservoirs and ion transport pathways, while micropores enhance the electrical double-layer capacitance by storing more electrolyte ions [61]. Thus, it is of great importance to optimize pore size to achieve high-performance supercapacitors.

**Figure 6:**
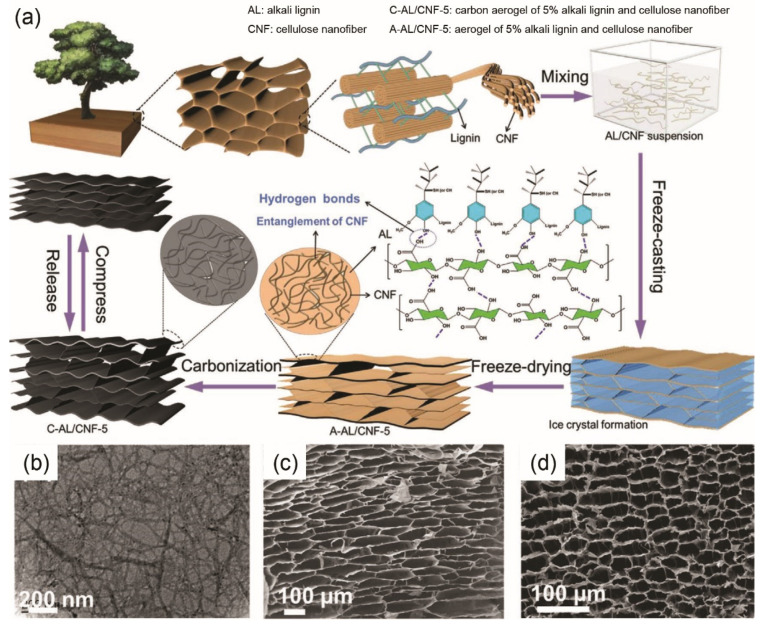
(**a**) Illustration of fabricating wood-derived C-AL/CNF-5; (**b**) Transmission electron microscope (TEM) image of CNF; Scanning Electronic Microscope (SEM) images of (**c**) A-AL/CNF-5 and (**d**) C-AL/CNF-5. C, A, AL, CNF represent carbon aerogel, aerogel, alkali lignin, cellulose nanofiber, respectively, and 5 indicates that the concentration of AL and CNF is 5%. (**a**–**d**) Reproduced with permission from ref. [62] Copyright 2020 John Wiley and Sons.

Lignocellulosic biomass materials have been widely used in the fabrication of 3D carbon aerogel electrode-based supercapacitors. For instance, wood-derived carbon aerogel, which was obtained by freeze-casting, freeze-drying and carbonization, demonstrated good compressibility (Figure 6a) [62]. The lignin and cellulose nanofiber-containing (Figure 6b) aerogel had a highly interconnected porous structure before (Figure 6c) and after (Figure 6d) carbonization, which was conducive to high EC performance. More details will be revealed in Section 5 regarding the EC performance of 3D carbon aerogel supercapacitors with electrodes derived from lignocellulose. Despite carbon aerogel’s unique porous structure, its lower flexibility or mechanical properties in comparison with 1D fiber or 2D film/fabric can be an obstacle that hinders its wide application in flexible supercapacitors.

## 4. Fabrication of Lignocellulose-Based Carbon

**Figure 7:**
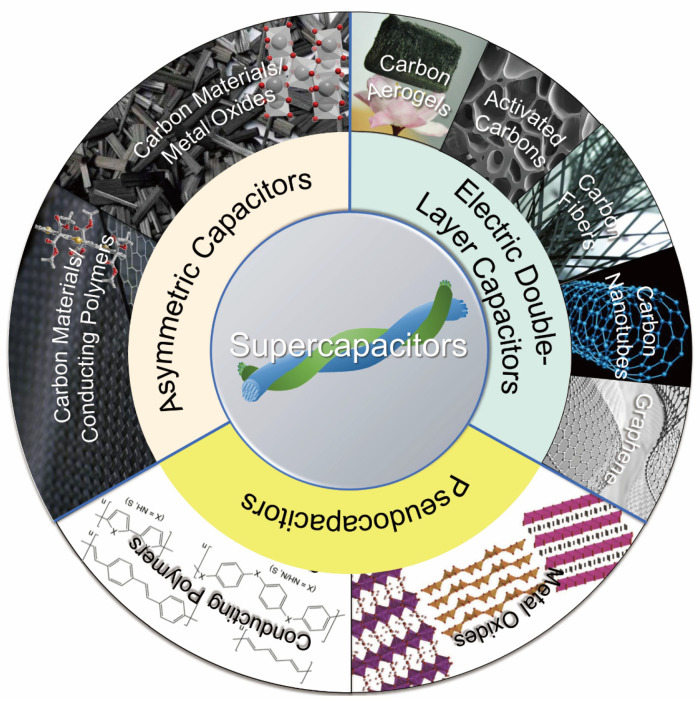
The diagram illustrating typical electrode materials for different types of supercapacitor. Carbon aerogels, activated carbons, carbon fibers, carbon nanotubes and graphene are mainly for EDLCs; metal oxide and conducting polymers are used for pseudocapacitors; carbon materials/conducting polymers or carbon materials/metal oxides are for asymmetric capacitors.

Electrode material is one of the key factors determining the EC performance of supercapacitors. Among various electrode materials (i.e., CNTs, AC, metal oxides, carbon fibers, conductive polymers, etc.) (Figure 7), carbon materials (graphene, CNTs and AC) [32,63,64] are widely used in EDLCs and asymmetric supercapacitors [65] mainly due to their desirable chemical and physical properties such as tunable porous structure, large SSA, excellent electrical conductivity, and good chemical/thermal stability [66].

One of the challenges to achieve carbon materials of good quality is to select a suitable source. The carbon source affects the structure or performance of the resulting carbon, including heteroatom content, nanostructure, and SSA [67,68]. Commercial AC, CNTs and rGO are the most commonly used carbon materials for supercapacitors. However, high production cost, potential environmental hazard, and complex and unsustainable preparation methods may hinder their wide application in flexible supercapacitors. Therefore, it is of great significance to develop biomass-derived carbon electrodes with long-term sustainability and low cost to achieve flexible supercapacitors with excellent EC performance.

**Figure 8:**
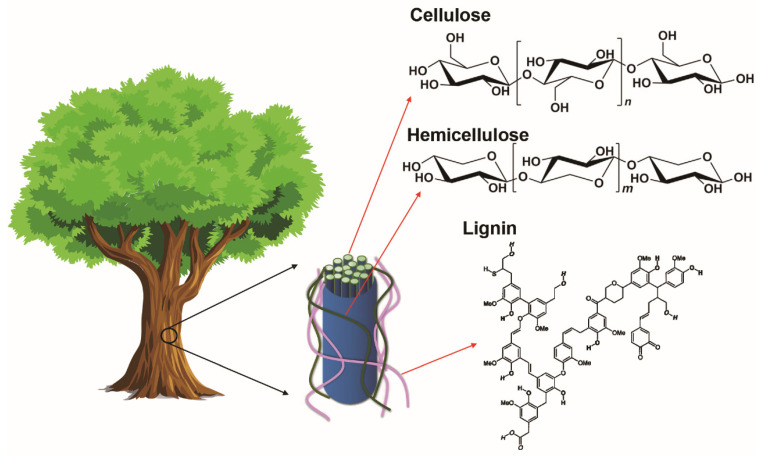
Cellulose strands are surrounded by hemicellulose and lignin in wood cell wall, and the corresponding chemical structures of carbohydrates (reproduced with permission from ref. [69] Copyright 2016 Elsevier) and lignin (reproduced with permission from ref. [70] Copyright 2002 John Wiley and Sons).

Lignocellulosic biomass consists of three main biopolymers, which are carbohydrates (cellulose and hemicellulose) and lignin (Figure 8). In the cell wall, the primary component is cellulose, which forms highly oriented and long fibrils. The amorphous, highly branched hemicellulose are attached to the adjacent cellulose fibrils by non-covalent crosslinks. Lignin is an amorphous, three-dimensional molecule linked to carbohydrates by hydrogen and covalent bonds. Typically, cellulose, lignin or raw lignocellulosic biomass are converted into carbon electrodes for flexible supercapacitors by carbonization, activation and possible surface modification. The SSA, porosity and surface chemistry can be tuned to yield flexible supercapacitors with good EC performance. These fabrication processes will briefly be introduced in this section.

### 4.1. Carbonization

Different methods have been developed to prepare biochar derived from lignocellulosic biomass. Carbonization of lignocellulosic biomass can be undertaken beforehand or combined with activation process. The thermochemical carbonization process, including pyrolysis and hydrothermal carbonization, was the earliest method used for the fabrication of biochar from biomass [71,72,73]. Pyrolysis occurs in the temperature range of 400–850 °C in an anoxic environment or an inert atmosphere to allow thermochemical decomposition of biomass for biochar. The properties of the resulting biochar mainly depend on the parameters such as heating rate, reaction temperature, catalyst and biomass type, etc. Hydrothermal carbonization is defined as the physical and chemical transformation of biomass in an aqueous environment with a low temperature (180–250 °C) and a saturated pressure (2–10 MPa) in a confined space. Dehydration, polymerization and carbonization occur in this process. The chemical structure, porosity and composition of the biochar can be tuned by carbonization parameters such as pressure, reaction time, catalyst and temperature, etc. However, the biochar or carbon material obtained by solely carbonization has limited SSA and porosity (Table 1), which are not preferred in the enhancement of the electrode’s EC performance [17]. Therefore, an activation process is always employed to increase the SSA and porosity of biochar [71].

### 4.2. Activation

Activation is an essential step to transforming lignocellulose-derived carbon into highly porous carbon materials for further use in flexible supercapacitors. It can be categorized into physical activation, chemical activation, and other methods such as physicochemical activation, microwave-assisted activation, and template-assisted activation, which will be introduced in this section. Table 2 summarizes the pore characteristics of lignocellulose-derived carbons based on different activation methods and activators.

#### 4.2.1. Physical Activation

In a physical activation process, the biochar is mainly activated by water (H_2_O) steam and carbon dioxide (CO_2_) in the temperature range of 700–1100 °C after carbonization [26,78,79,80]. The chemical reactions between biochar and H_2_O or CO_2_ in a physical activation process are as expressed in the following Equations (1)–(3) [81,82]:

(1) $$C+H_{2}O\to \mathrm{CO}↑+H_{2}↑$$

(2) $$C+{\mathrm{CO}}_{2}\to 2\mathrm{CO}↑$$

(3) $$\mathrm{CO}+H_{2}O↔{\mathrm{CO}}_{2}↑+H_{2}↑$$

The activation agents promote high SSA and high total pore volume by controlled active carbon atom burn-off and elimination of volatile substances [26]. The porosity or the quality of the activated lignocellulose-derived carbon relies on different activation parameters such as temperature, time, gas flow rate, etc. [26,28]. Moreover, an appropriate activation agent is required for biomass to achieve high surface area or porosity due to the difference in chemical structure and composite component of precursor [83]. For instance, pine nutshell activated by H_2_O steam at 800 °C had SSA of 956 m^2^/g, total pore volume of 0.62 cm^3^/g and a mesopore ratio of 37.1% [84]. CO_2_ was employed as an activator of willow at 800 °C to yield lignocellulose-based carbon with SSA of 739 m^2^/g and total pore volume of 0.37 cm^3^/g [85]. However, it is quite hard to achieve SSA > 2000 m^2^/g with physical activation. Thus, an alternative activation method such as chemical activation can be used to obtain lignocellulose-derived carbon with abundant micropores and high SSA.

#### 4.2.2. Chemical Activation

In a chemical activation process, the biochar is pre-mixed with chemical activation agent like a strong base (i.e., potassium hydroxide (KOH) [86,87], sodium hydroxide (NaOH) [88]), an acid (i.e., H_3_PO_4_ [89,90]), or a salt (i.e., zinc chloride (ZnCl_2_) [91]). Then the mixture is subjected to simultaneous carbonization/activation at an elevated temperature (300–900 °C), which affects pyrolytic decomposition and results in highly porous structure [26].

Among different chemical activation agents, KOH is the most widely used activation agent since it demonstrates the best activation effect to enhance the SSA up to >2000 m^2^/g [92,93,94]. The mechanism of KOH activation involves the etching of carbon skeleton for the expansion or creation of pores caused by the reactions at high temperature (>750 °C), as shown in Equations (4)–(7) [75,95]:

(4) $$2\mathrm{KOH}+{\mathrm{CO}}_{2}\to K_{2}{\mathrm{CO}}_{3}+H_{2}O↑$$

(5) $$2C+2\mathrm{KOH}\to 2\mathrm{CO}↑+2K↑+H_{2}↑$$

(6) $$K_{2}{\mathrm{CO}}_{3}+C\to K_{2}O+2\mathrm{CO}↑$$

(7) $$K_{2}O+C\to 2K↑+\mathrm{CO}↑$$

Different types of lignocellulose show various pore characteristics after chemical activation with KOH [94,96,97,98]. For example, walnut shell activated by KOH had extremely high SSA of 3577 m^2^/g and large pore volume of 2.19 cm^3^/g [96]. Lignocellulose-based carbon from tobacco rods by hydrothermal carbonization and KOH activation also possessed high SSA of 2115 m^2^/g and pore volume of 1.22 cm^3^/g [94].

NaOH is another commonly used activation agent for lignocellulose-derived AC. It has less corrosiveness, lower weight, and lower cost [99], in comparison with the aforementioned KOH. For instance, wheat bran-derived carbon by NaOH activation at 800 °C demonstrated substantial SSA (2543 m^2^/g) and high total pore volume (1.68 cm^3^/g) [100]. The biochar from peanut shell activated by NaOH had superior SSA of 2764 m^2^/g and large total pore volume (1.31 cm^3^/g) [101]. 

As a mild salt activation agent, ZnCl_2_ is also a frequently used activation agent to yield biomass-derived porous carbon. The possible activation mechanism of ZnCl_2_ is based on the catalytic dihydroxylation and dehydration during pyrolysis process. In this process, H and O atoms are released as H_2_O, creating a micropore-rich structure in biochar [28]. Moreover, ZnCl_2_ acts as a framework for carbon deposition in the activation process, and leaves voids in carbon skeleton for more pores after acid washing [28]. For example, waste tea activated by ZnCl_2_ at 450 °C displayed good SSA of 1308 m^2^/g and total pore volume of 0.81 cm^3^/g.

In comparison with physical activation, chemical activation has shorter reaction time, lower reaction temperature, and yields activated biochar with higher SSA and total pore volume. However, it is necessary to wash the activation carbon to remove chemical reagent residues and ash content after chemical activation. Thus, this process has some drawbacks such as high cost, equipment corrosion and non-recyclable chemicals.

#### 4.2.3. Other Activation Methods

Other activation methods such as physicochemical activation, microwave-assisted activation, and template-assisted self-activation have been employed in the activation of lignocellulose-derived carbon materials (Table 2).

Physicochemical activation is a combination of physical and chemical activation to yield AC with good porosity by partially replacing hazardous chemicals with steam [28]. For instance, peanut shell activated by bimetallic activation with CO_2_ at 800 °C yielded biochar with SSA of 1428 m^2^/g, total pore volume of 0.55 cm^3^/g, and a large proportion of micropores (73.91%) [102]. Lignin activated by KOH/H_2_O with microwave heating showed high SSA (2482 m^2^/g) and total pore volume (1.45 cm^3^/g) [103]. Microwave-assisted activation transforms the microwave energy with dipole rotation and ionic conduction within the particles [104] to yield biomass-based AC. Lignin activated by KOH with microwave had significant SSA of 3065 m^2^/g and pore volume of 0.54 cm^3^/g [105]. Microwave-assisted activation of coconut shell by CO_2_ also showed good pore characteristics with SSA of 2288 m^2^/g and pore volume of 1.3 cm^3^/g [106].

Template-assisted self-activation converts biomass into AC with no additional activators. The biomass precursor’s inherent inorganic salts or metallic ions could etch the carbon skeleton or react with pyrolysis gases (i.e., CO_2_, H_2_O) generated in the carbonization process [107,108]. This method combines the template and self-activation agents together without post-activation [109]. For example, cotton containing magnesium oxide (MgO) and ZnCl_2_ was converted into AC with MgO as template and ZnCl_2_ as activator. The resulting biochar exhibited satisfactory SSA of 1990 m^2^/g and pore volume of 1.23 cm^3^/g [110].

**Table 2:** Examples of lignocellulosic carbon prepared by different carbonization/activation methods.

Carbonization	Activation	Activation Agent	Raw Material and Reference	Pore Characteristics	
				SSA (m^2^/g)	Pore Volume (cm^3^/g)
Pyrolysis	Physical activation	H_2_O steam	Pine nutshell [84]	956	0.62
		CO_2_	hybrid willow [85]	739	0.37
	Chemical activation	KOH	Walnut shell [96]	3577	2.19
			Wheat straw [97]	2560	1.17
		NaOH	Peanut shell [101]	2764	1.31
			Wheat bran [100]	2543	1.68
		H_3_PO_4_	Switchgrass [111]	1373	1.44
		ZnCl_2_	Waste tea [112]	1308	0.81
	Physichemical activation	KOH/H_2_O steam	Lignin [103]	2482	1.45
		FeCl_3_ + ZnCl_2_/CO_2_	Peanut shell [102]	1428	0.55
	Microwave-assisted activation	KOH	Lignin [105]	3065	0.54
		CO_2_	Coconut shell [106]	2288	1.30
	Template-assisted self-activation	MgO/ZnCl_2_	Cotton [110]	1990	1.23
Hydrothermal	Chemical activation	KOH	Cypress coats [98]	1326	0.78
			Tobacco rods [94]	2115	1.22
	Microwave-assisted activation	KOH	Distiller’s dried grains with solubles [113]	479	~0.15

H_2_O: water; CO_2_: carbon dioxide; KOH: potassium hydroxide; NaOH: sodium hydroxide; H_3_PO_4_: phosphoric acid; ZnCl_2_: zinc chloride; FeCl_3_: ferric chloride; MgO: magnesium oxide.

In summary, lignocellulose-derived AC materials typically have the following common characteristics: (a) large SSA to supply sufficient active sites for electrolyte ions storage [114,115]; (b) well-developed porous structure to facilitate fast diffusion of electrolyte ions into pores at high current loads [116,117]; (c) a 3D interconnected porous framework to ensure fast electrolyte ion transfer [118,119]; (d) a heteroatom-rich structure to improve surface wettability and offer additional pseudocapacitance [120]. Thus, they are suitable and promising electrode materials to fabricate flexible supercapacitors with high EC performance.

### 4.3. Surface Modification

The EC performance of carbon materials for supercapacitors is mainly dictated by the architecture of the porous structure [17] and the surface chemistry. Three-dimensional (3D) hierarchically porous structure with different portions of micro-, meso- and macropores can be achieved by proper selection and control of the activation as mentioned above. However, the surface chemistry of AC is not affected by the chosen activation process [121]. The surface modification of biochar generally changes the electron accepting/donating characteristics, and the chemical structures. The EC performance is influenced by the introduction of heteroatoms (i.e., O, H, N, S, P, etc.). The acidic or basic characteristic of the AC surface is determined by the surface functional groups and the delocalized electron of the carbon structure [122]. Acidic surface groups are related to oxygen-containing functionalities, such as carboxylic, lactonic, and phenolic groups, which are unstable and can be obtained by the exposure of carbon to oxygen at high temperature or by the reaction of carbon with oxidation agents at room temperature [123]. A basic surface feature originates from the delocalized π-electrons of carbon structure or basic surface functionalities such as nitrogen-containing groups [124]. Thus, one efficient way to increase the number of basic surface groups of AC is to either remove/neutralize the acidic functional groups, or to replace acidic groups with basic groups. To properly tailor the surface chemistry of the activated biochar, surface modification is achieved by thermal or chemical treatments.

Thermal treatment refers to the heat treatment of AC at high temperature (700–1000 °C) in hydrogen (H_2_) or inert atmosphere via conventional or microwave heating. The main purpose of thermal treatment is to selectively decompose or remove some oxygen-containing surface functionalities to achieve basic characteristics [125]. Thermal treatment is completed in H_2_ or an inert atmosphere (i.e., nitrogen (N_2_) or helium (He)) to yield oxygen-free surface [26,124]. H_2_ is very effective in the removal or decomposition of the oxygen functionalities, the stabilization of the carbon surface by deactivating the active sites via the formation of carbon–hydrogen (C–H) bonds, and the gasification of unstable and reactive carbon atoms [124].

Chemical surface modification of AC can be achieved by the addition of chemical agents (i.e., nitric acid (HNO_3_), hydrogen peroxide(H_2_O_2_), sulfuric acid (H_2_SO_4_) [126,127], ammonium peroxydisulfate, melamine [128] and ammonia (NH_3_) [129], etc.) to introduce acidic (oxygen-containing) or basic (nitrogen-containing) functional groups. For instance, carbon atoms on the AC surface can be partially oxidized by HNO_3_ to yield oxygen-containing functional groups such as hydroxyl (-OH), and carboxyl (-COOH) groups [126]. These functional groups further participate in redox reactions to offer extra pseudocapacitance. They also boost the polarity and hydrophilicity of AC, which contribute to the enhanced EC performance of the AC-based electrode. However, excessive oxygen-containing surface groups can hinder the organic electrolyte diffusion into the pores and deteriorate the capacitive performance of the AC electrode [126]. Nitrogen-rich (graphitic-, pyridinic-, pyrrolic- and pyridinic-N) AC showed better EC performance than the oxygen-rich one. In general, pyridinic-N and graphitic-N facilitate the acceleration of the electron transfer, while electrochemically active and electron-rich nitrogens (pyridinic-N and pyrrolic-N) provide pseudocapacitance effects [128,130,131].

In summary, heteroatoms determine the chemical properties of AC, and influence the EC performance of the assembled supercapacitors [132]. In particular, heteroatoms provide additional pseudocapacitance, increase electrical conductivity, promote charge transfer and alter surface wettability to facilitate the electrode/electrolyte interface reactions. Lignocellulose-derived AC inherits the heteroatoms from the precursor materials in the chemical structure after controlled carbonization. Also, these atoms can be introduced into AC by heteroatom-containing dopants during the activation process [17].

## 5. Electrochemical (EC) Performance of Lignocellulose-Derived Carbon-Based Flexible Supercapacitors

The heteroatoms that originate from the chemical structure of lignocellulosic biomass precursor materials, together with 3D hierarchically porous structure in lignocellulose-derived AC, often result in the enhancement of capacitance performance of supercapacitors. In this section, EC performance of flexible supercapacitors with cellulose-, lignin- and readily available lignocellulose-based carbon electrodes will be discussed.

### 5.1. Flexible Supercapcitors from Cellulose-Derived Carbon

Cellulose plays an indispensable role in the preparation of active electrode materials of flexible supercapacitors due to good flexibility, light weight, low cost and sustainability [133,134]. Table 3 summarizes different aspects of cellulose-based carbon for flexible supercapacitors including raw material based on cellulose or its derivatives, thermal treatment condition, pore characteristics and EC performance, which will be introduced in this section.

One-dimensional (1D) cellulose fiber-based carbon electrode can be achieved by wet-spinning followed by carbonization. However, literature in this area is quite limited, which is possibly due to the high cost of cellulose fiber spinning. Nitrogen-doped, cellulose nanofiber-based flexible carbon mesh was prepared by the computerized numerical control of wet-spinning, and the carbonization of cellulose/graphene oxide (GO)/silk fibroin (mass ratio of 55/40/5) mixture [135]. The resulting carbon mesh had wrinkled morphology of the carbon fiber structural unit, which was oriented along the axial direction. After nitrogen-doping, the interconnected mash structure facilitated the electron movement, increased the transport path of electrons, offered extra pseudocapacitance and large SSA, thereby significantly enhancing the EC performance of the carbon mesh electrode-based flexible symmetric supercapacitor: maximum areal specific capacitance of ~4.5 mF/cm^2^ at a current density of 2.5 μA/m^2^, and excellent folding endurance with capacitance retention up to 152% after 50,000 folding times.

Carbon nanofibers obtained by carbonization of partially hydrolyzed cellulose acetate nanofibers showed great potential as electrode materials to fabricate flexible supercapacitors with high specific capacitance of ~241.4 F/g at 1 A/g [136]. The significant EC performance is ascribed to the inter-bonded fibrous structure and porous nanofiber surface. The relatively high SSA, appropriate pore distribution, good electric conductivity, short ion diffusion path, and fast charge transfer rate within the electrode during charge/discharge also contribute to the EC performance. Carbonized cellulose paper composing of carbon fibers was synthesized via nickel (Ni) decoration and subsequent carbonization. The free-standing Ni-decorated carbon paper demonstrated high specific capacitance of 268 F/g at 0.2 A/g [137]. Thus, flexible, highly conductive 2D cellulose-based electrodes with good EC performance are promising for the fabrication of high-performance flexible supercapacitors.

**Figure 9:**
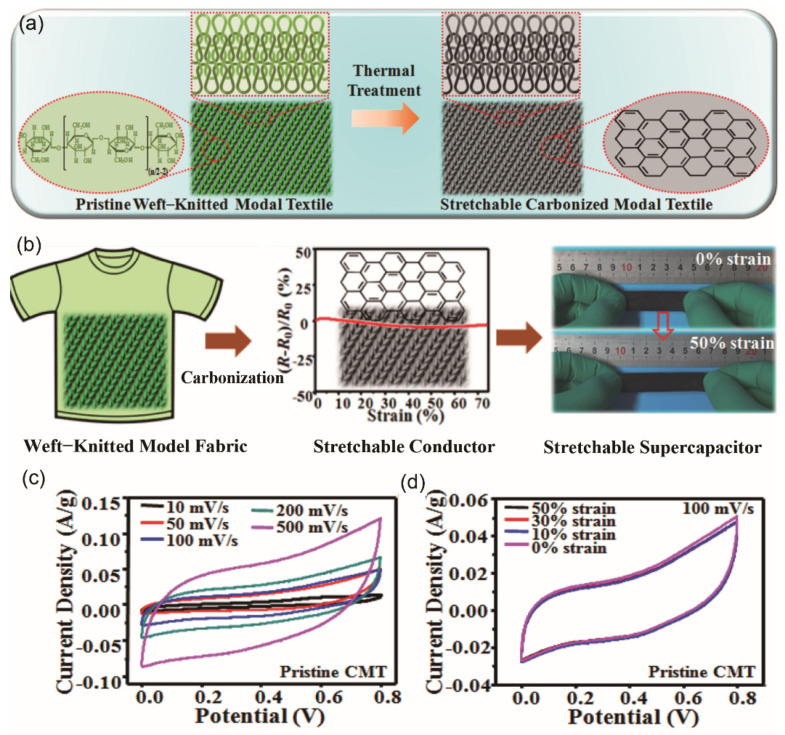
(**a**) Schematic illustration showing the transformation of a Modal textile into a graphite-like carbon textile through a thermal treatment process. (**b**) Fabrication of a stretchable supercapacitor using weft-knitted Modal fabric as the electrodes. (**c**) Cyclic voltammetry (CV) curves of the supercapacitor at different scan rates. (**d**) CV curves of the supercapacitor at a scan rate of 100 mV/s and under strains of 0%, 10%, 30%, and 50%. (**a**–**d**) Reproduced with permission from ref. [58] Copyright 2017 American Chemical Society.

Cellulose fibers have been widely used in the manufacturing of textile fabrics. As mentioned in Section 3.2, inherently stretchable and conductive fabric-based supercapacitors were fabricated from cellulose based Modal raw material by weft-knitting technology, followed by carbonization [58]. The assembled stretchable supercapacitor demonstrated stable EDLC behavior and unique mechanical-electrical properties (Figure 9). The EC performance was quite mediocre and it could be optimized by employing higher carbonization temperature to decrease the internal resistance of carbonized fabric or by increasing the SSA in the carbonization process.

Carboxymethyl cellulose (CMC) was coated on cotton fabric, and later a designed pattern of carbonized CMC was obtained with laser direct writing process. Metal oxide (M_x_O_y_)/carbonized CMC electrodes on the substrate fabrics were separated by a layer of PVA/H_3_PO_4_ electrolyte to form supercapacitors with a capacitance of 1.095 mF/cm^2^, high flexibility and mechanical durability [138]. However, the durability of coated material, and the adhesion between active electrode and substrate fabric remain to be further investigated. To achieve good adhesion, cellulose textile fabric integrated with polydopamine was obtained by in situ polymerization of dopamine on the fabric [139]. The carbonized fabric surface was covered by interconnected polydopamine nanoparticles that formed a 3D porous network. The porous and interconnected structure offered high SSA (347.6 m^2^/g) and facilitated fast ion transportation. As a result, the all-solid-state supercapacitor based on the flexible fabric electrode had excellent capacitance of 1208 mF/cm^2^, as well as excellent flexibility.

Cellulose-based 3D carbon aerogels are also promising electrode materials for flexible supercapacitors due to their versatile properties (i.e., sustainability, high porosity, good flexibility, large SSA, high electrical conductivity and 3D porous structure) [140,141,142,143]. For example, they could be prepared by dissolving microcrystalline cellulose in NaOH aqueous solution, followed by gelation, regeneration, and supercritical CO_2_ drying [144]. After pyrolysis and subsequent CO_2_ activation, cellulose-based activated carbon aerogels exhibited a high SSA of 1873 m^2^/g and a large pore volume of 2.65 cm^3^/g. The flexible supercapacitors possessed promising performance: specific capacitance values of 302 F/g and 205 F/g at 0.5 A/g and 20 A/g, respectively, and capacitance retention of 92% after 4000 cycles of charge/discharge. Similarly, Zhou et al. [145] synthesized hierarchically porous cellulose-based carbon aerogel by dissolving-gelling and freeze-drying processes. After carbonization and CO_2_ activation, 3D carbon aerogel with abundant macro-, meso- and micropores displayed high specific capacitance of 328 F/g at 0.5 A/g, and 96% of capacitance retention after 5000 cycles. As mentioned, hierarchical porous structures with micro-, meso- and macropores are effective to achieve fast ion transport, shorten ion transport pathways, and store more electrolyte ions to enhance the electrical double-layer capacitance [61]. KOH-activated carbon aerogel from sodium CMC had a capacitance of 152.6 F/g at 0.5 A/g. Its highly porous and 3D interconnected structure results in better conductivity and ion transportation [146]. Moreover, all-nanofiber asymmetric supercapacitors with nanocellulose-derived hierarchically porous carbon anode (SSA of 2046 m^2^/g), a mesoporous nanocellulose membrane separator, and a nickel cobaltite (NiCo_2_O_4_) cathode with nanocellulose carbon as the support matrix were fabricated. The supercapacitors with 3D porous structure had capacitance of 64.83 F/g at 0.25 A/g, and energy density of 23.05 Wh/kg at 213 W/kg [147].

Carbon aerogels based on wood-derived cellulose nanofibers have been synthesized by pyrolysis with p-toluene sulfonic acid (TsOH) catalyst. The electrode had an electrically conductive 3D nanofibrous network structure, and a large SSA (689 m^2^/g), thereby a high specific capacitance of 140 F/g at 0.5 A/g [148]. Nitrogen-doped carbon nanofibers/graphene aerogels were prepared from cellulose nanofibers/graphene oxide (GO)/ethylenediamine mixture by freeze-drying and carbonization. The device exhibited a capacitance of 289 F/g at 5 mV/s and a capacitance retention of 90.5% [149]. It is believed that the porous structure of the carbon aerogel with high porosity offers short ion transfer pathways during charge/discharge. In addition, the good electrical conductivity and wettability due to nitrogen-doping facilitate the electron transport and electrolyte permeation, further contributing to the enhanced EC performance [149]. Elastic carbon aerogel with cellulose nanofibers and lignin was fabricated as a flexible electrode by freezing-casting, vacuum freeze-drying, and carbonization [62]. The highly entangled flexible cellulose nanofibers easily formed an interconnected framework, whereas lignin provided sufficient mechanical stiffness. The flexible and free-standing all-solid-state symmetric supercapacitor was assembled with two cellulose/lignin aerogel electrodes, a PVA/H_2_SO_4_ gel electrolyte and a cellulose paper separator in a sandwich structure. The supercapacitor had good mechanical flexibility and mediocre EC performance (specific capacitance of 62.4 F/g at 0.5 A/g, rate capability of 58.5% from 0.5 A/g to 4.0 A/g and energy density of 8.6 Wh/kg at power density of 250 W/kg) (Table 3). Despite high SSA (1124 m^2^/g), the tracheid-like structure of the carbon aerogels (Figure 6) demonstrated dominant large pores, and insufficient micro- and mesopores, which resulted in mediocre EC performance.

**Table 3:** Literature summary of cellulose-based carbon for flexible supercapacitors.

Cellulose-Derived Carbon/Other Material and Reference	Thermal Treatment	Porosity Characteristics	Electrotyle	Electrochemical (EC) Performance
Electropsun partially hydrolyzed cellulose nanofibers-derived carbon [136]	Stabilization: 240 °C, 3 °C/min, air Isothermal treatment: 240 °C, 1 h, N_2_ Cabonization: 1000 °C, 5 °C/min, 2 h Activation with CO_2_: 850 °C, 1.5 h	SSA: 530–700 m^2^/g Pore volume: 0.30–0.4 cm^3^/g	6 M KOH	C_s_: 241.4 F/g at 1 A/g Cycling stability 99.9%, 10,000 cycles Maximum power density: 84.1 kW/kg
Cellulose paper-derived carbon decorated with Ni [137]	Carbonization: 700 °C, 2 h, N_2_	SSA: 130–369 m^2^/g Pore volume: 0.07–0.13 cm^3^/g	6 M KOH	C_s_: 268 F/g at 0.2A/g Capacitance retention: 97%, 2000 cycles
Nitrogen-doped cellulose nanofiber-derived carbon/graphene aerogel [149]	N/A	N/A	1 M H_2_SO_4_	C_s_: 289 F/g at 5 mV/s Cyclic stability: 90.5%, 5000 cycles
Ultrathin cellulose nanofiber-derived carbon aerogel [148]	Stepwise carbonization: 500 °C, 2 °C/min, 1 h; 800 °C, 5 °C/min, 2 h, Ar, TsOH	SSA: 689 m^2^/g Pore size: 0.2–0.9 nm	0.2 M H_2_SO_4_	C_s_: 140 F/g at 0.5 A/g Cyclie stability: 90.5%, 5000 cycles Power density: 48,600 W/kg Cyclic stability: 100%, 10,000 cycles
Cellulose/GO/silk fibroin-derived (mass ratio of 55/40/5) wet-spun carbon fiber mesh [135]	Carbonization: 400 °C, 1 °C/min; 800 °C, 5 °C/min, 2h, Ar/H_2_	N/A	PVA/H_3_PO_4_	C_s_: 4.5 mF/cm^2^ at 2.5 μA/m^2^ Capacitance retention: 152%, 50,000 folding times
Commercially available Modal knitted fabric-derived carbon cloth [58]	Carbonization: 600 °C, 700 °C, 800 °C, 900 °C, and 1050 °C, 3 °C/min, 200 min, Ar/H_2_	N/A	PVA/H_3_PO_4_	C_s_: 7.5 mF/cm^2^ (1.2 F/g) at 10 mV/s Capacitance retention: 44%, from 50 mV/s to 500 mV/s
CMC/M_x_O_y_-derived carbon on cotton fabric [138]	Laser direct writing with laser power 0.25–0.35 W and scanning velocity 2.5 mm/s	SSA: 22.13–37.12 m^2^/g	PVA/H_3_PO_4_	C_s_: 1.095 mF/cm^2^ Cyclic performance: 92%, 1000 cycles
Cellulose textile fabric/polydopamine-derived carbon fabric [139]	Stepwise carbonization: 300 °C, 5 °C/min, 1 h, N_2_; 1000 °C, 5 °C/min, 1 h, N_2_	SSA: 347.6 m^2^/g	PVA/LiCl	C_s_: 1208.4 mF/cm^2^ (10.1 F/cm^3^) at 1 mA/cm^2^ Cyclic performance: 94%, 4000 cycles
Microcrystalline cellulose-derived carbon aerogel [144]	Carbonization: 800 °C, 5 °C/min, 2 h, N_2_ Activation: 800 °C, 3 h, N_2_; 4 h, CO_2_	SSA: 1873 m^2^/g Total pore volume: 2.65 cm^3^/g	6 M KOH	C_s_: 302 F/g at 0.5 A/g; 205 F/g at 20 A/g Capacitance retention: 92%, 4000 cycles
Cellulose-derived carbon aerogel [145]	Stepwise carbonization/activation: 200 °C, 5 °C/min, 2 h; 800 °C, 3 °C/min, 2 h, CO_2_	SSA: 1346 m^2^/g Total pore volume: 1.42 cm^3^/g	1 M H_2_SO_4_	C_s_: 328 F/g at 0.5 A/g Capacitance retention: 96%, 5000 cycles
Sodium CMC-derived carbon aerogel [146]	Stepwise carbonization: 550 °C, 10 °C/min; 900 °C, 5 °C/min, 2 h, N_2_ Activation with KOH: 900 °C, 10 °C/min, 1 h, 2 h, and 3 h, N_2_	SSA: 428 m^2^/g Total pore volume: 0.35 cm^3^/g	6 M KOH	C_s_: 152.6 F/g at 0.5 A/g
Nanocellulose-derived hierarchical porous carbon anode [147]	Carbonization/activation with ZnCl_2_: 750 °C, 3 °C/min, 2 h, Ar	SSA: 2046 m^2^/g	6 M KOH	C_s_: 64.83 F/g (10.84 F/cm^3^) at 0.25 A/g; 32.78 F/g (5.48 F/cm^3^) at 4 A/g Energy density: 23.05 Wh/kg at power density of 213 W/kg
Carbon aerogel from alkali lignin/cellulose nanofiber (mass ratio 1/1) [62]	Carbonization: 800 °C, 3 °C/min, 2 h	SSA: 1124 m^2^/g Pore volume: 1.95 cm^3^/g	PVA/H_2_SO_4_	C_s_: 62.4 F/g at 0.5 A/g (231 mF/cm^2^ at 0.5 mA/cm^2^) Rate capability: 58.5%, from 0.5 A/g to 4.0 A/g Capacitence retention: 88.5%, 5000 cycles Energy density: 8.6 Wh/kg at power density of 250 W/kg

N/A: not available; SSA: specific surface area; GO: graphene oxide; PVA: poly (vinyl alcohol); KOH: potassium hydroxide; H_2_SO_4_: sulfuric acid; H_3_PO_4_: phosphoric acid; CMC: carboxymethyl cellulose; C_s_: specific capacitance; Ni: nickel; CO_2_: carbon dixoide; N_2_: nitrogen atmosphere; H_2_: hydrogen atmosphere; Ar: argon atmosphere; LiCl: lithium chloride; M_x_O_y_: metal oxide; ZnCl_2_: zinc chloride; TsOH: p-toluene sulfonic acid.

**Figure 10:**
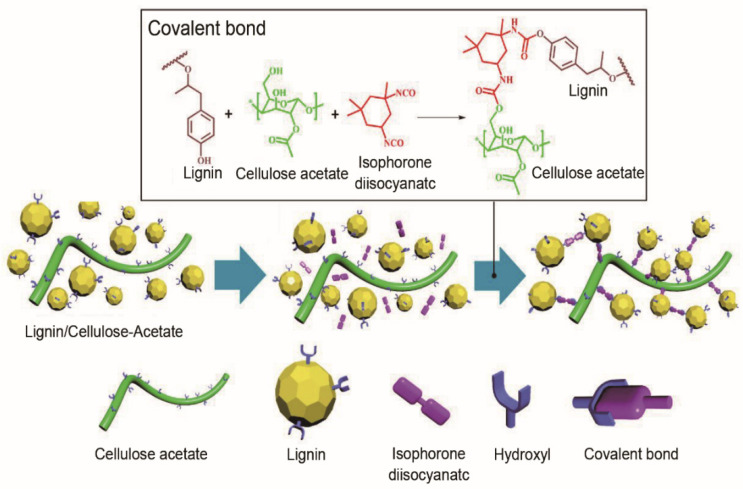
Preparation mechanism of modification reaction with lignin and cellulose acetate. Reproduced with permission from ref. [151] Copyright 2019 American Chemical Society.

Apart from the aforementioned cellulose products used for carbon electrodes of flexible supercapacitors, commonly used carbon precursors of flexible supercapacitors include cellulose derivatives such as cellulose esters, cellulose ethers and cellulose ether esters. These are functional polymers obtained by the esterification or etherification of cellulose hydroxyl functional groups with chemical reagents [150]. Cellulose derivatives are mainly employed as substrates for flexible supercapacitors. Studies of active materials for flexible/wearable energy storage devices based on these cellulose derivatives are still at an initial stage. For instance, isophorone diisocyanate (IPDI) was used as a chemical modifier to effectively connect lignin and cellulose acetate by covalent bond (Figure 10) for better fiber spinnability [151]. The biomass-based carbon fibers had enhanced mechanical properties (maximum tensile strength of 49 MPa, Young’s modulus of 3 GPa), as well as good EC performance (later presented in Section 5.2, Table 4). Cellulose acetate was mixed with phosphatized lignin to fabricate an electrospun biomass-derived carbon electrode [152,153]. The optimal specific capacitance of biomass-derived carbon nanofibers as a three-electrode system was 363.1 F/g. At a power density of 800 W/kg, the biomass-based supercapacitor device delivered an energy density of 31.2 Wh/kg. The electrodes exhibited great potential for a flexible free-standing supercapacitor with capacitance retention of 98% after 10,000 cycles. It is believed that molecular spatial conformation of lignin plays a crucial role in improving the properties of biomass-based carbon electrodes. Thus, it is important to understand the relationship between the structure/functionality of lignocellulose, the lignocellulose-derived carbon electrode and the EC performance of supercapacitor.

In summary, the aforementioned reports have shown that cellulose-derived carbon is a promising material towards green and high-performance flexible SCs with facile preparation process and hierarchical porous structures.

### 5.2. Flexible Supercapcitors from Lignin-Derived Carbon

Lignin-derived carbon fibers possess high SSA, short diffusion paths, and a rich porous structure that facilitate faster electron conduction and more efficient electrolyte infiltration in flexible supercapacitors. Thus, lignin is a promising carbon precursor to yield microporous, oxygen-functionalized activated carbon fibers (ACFs) [154] or carbon nanofibers (CNFs) electrodes with good EC performance [155,156,157,158,159,160]. Electrospun Kraft lignin-based ACFs were prepared by low temperature stabilization (250 °C), carbonization (900 °C for 1 h in N_2_), and CO_2_ activation (800 °C for 1 h). ACFs electrode had specific gravimetric capacitance of 155 F/g at 0.1 A/g, excellent rate capability with 113 F/g at 250 A/g and good capacitance retention of 94% after 6000 cycles [155]. Similarly, lignin-based ACFs were fabricated with hexamine in spinning solution acting as crosslinker to accelerate the thermostabilization process [156]. The resulting ACFs electrode possessed good capacitance (133.3 F/g at a current density of 1 A/g), and high energy density and power density (42 Wh/kg and 91 kW/kg, respectively) due to large SSA (2185 m^2^/g) and porous structure. Moreover, the activation of electrospun lignin-based mats with H_3_PO_4_ in the presence of oxygen gas (O_2_) boosted SSA up to 2340 m^2^/g. The resulting ACFs electrode had an optimal specific capacitance of 240 F/g in 1 M H_2_SO_4_, and the assembled symmetric supercapacitors had specific capacitance of 48 F/g at 2 A/g, energy density of 8.4 Wh/kg, maximum power density of 47 kW/kg [157]. Hardwood and softwood lignins were used to develop different porosities after crosslinking and degradation upon high temperature treatment due to the difference in side-chain structure, which in turn influenced the EC performance of lignin-based CNFs in flexible supercapacitors. Hardwood lignin showed both a higher degree of crosslinking and degradation, hence developing higher SSA (~1600 m^2^/g) than that of softwood lignin (~1300 m^2^/g) Thus, the best performance of a free-standing electrode in supercapacitors was observed by hardwood lignin stabilized at 310 °C with specific capacitance of 164 F/g at 0.1 A/g and 119 F/g at 250 A/g, respectively, and a capacitance retention of >90% after 10,000 cycles [158].

**Table 4:** Electrospun lignin/polymer-derived carbon nanofibers as electrodes for flexible supercapactitors.

Lignin/Polymer (Mass Ratio) in Solvent/Additive and Reference	Thermal Treatment	Activation Agent	Porosity Characteristics	Electrotyle	Eelectrochemical Performance
Lignin/PAN (0/100, 40/60, 60/40, and 70/30) in DMF [161]	Stabilization: 250 °C, 1 °C/min, air Carbonization: 800 °C, 10 °C/min, 1 h, N_2_	N/A	SSA: 578–675 m^2^/g Total pore volume: 0.25–0.29 cm^3^/g	6 M KOH	C_s_: 216.8 F/g Capacitance retention: 88.8%, 2000 cycles
Lignin/PAN (0/100, 10/90, 20/80, 30/70) in DMF [162]	Stabilization: 280 °C, 2 °C/min, 1 h, air Carbonization: 1000 °C, 5 °C/min, 1 h, N_2_ Activation: 1000 °C, 1h, CO_2_	CO_2_	SSA: 1927–2543 m^2^/g Micropore volume: 0.486–0.697 cm^3^/g Macropore volume: 0.277–0.955 cm^3^/g	Pyr_14_TFSI ionic liquid/PC/EC(volume ratio of 3/3/2)	C_s_: 128 F/g Energy density: 59 Wh/kg at 1 A/g
Lignin/PAN (80/20, 50/50)/NiCo_2_O_4_ [163]	Stablization: 280 °C, 1 °C/min, 1 h, air Carbonization: 1000 °C, 5 °C/min, N_2_	N/A	N/A	Electrode: 2 M KOH SC: PVA/KOH	C_s_ of electrode: 1757 F/g at 2 mA/cm^2^; cycle stability: 138%, 5000 cycles C_s_ of device: 134.3 F/g at 1 A/g, energy density: 47.75 Wh/kg at power density of 799.53 W/kg
Lignin/PAN (50/50) in DMF/Ni-Mn sulfide [164]	Stablization: 240 °C, 1 °C/min, 4 h, air Carbonization: 800 °C, 4 °C/min, N_2_	N/A	SSA: 35.8 m^2^/g	Electrode: 1 M KOH Asymmetric SC: 2 M KOH	C_s_ of electrode: 652.3 C/g at 1 A/g; cycling stability: 91.3%, 5000 cycles at 10 A/g SC device: energy density of 52.4 Wh/kg at power density of 800 W/kg; capacitance retention: 92.3%, 10,000 cycles
Lignin/PAN (10/90) in DMF/MnCl_2_ [165]	Stablization: 280 °C, 1 h, air Carbonization: 800 and 900 °C, 5 °C/min, 1 h	N/A	N/A	6 M KOH	C_s_: 212 F/g Energy density: 26.5 Wh/kg at power density of 400 W/kg
Lignin/PAN/pitch (0.78/7/3) in THF or DMF/ZnO [166]	Stablization: 280 °C, 1 h, air Carbonization: 800 °C, 4 °C/min, 1 h, N_2_	N/A	SSA: 376–1194 m^2^/g	6 M KOH	C_s_: 165 F/g at 1 mA/cm^2^ Energy density: 22–18 Wh/kg at power density of 400–10,000 W/kg Cycle stability: 94%, 3000 cycles
Lignin/PAN (50/50) in DMF/nitrogen-sulfur co-doped graphene [167]	Stabilization: 260 °C, 0.5 °C/min, 3h, air Carbonization: 1400 °C, 5 °C/min, 1 h, N_2_ Activation: 800 °C, 10 °C/min, 1 h, N_2_	KOH	SSA: 1008–2439 m^2^/g	6 M KOH	C_s_: 267 F/g at 5 mV/s Energy density: 9.28 Wh/kg at power density of 493 W/kg Capacitance retention: 96.7%, 5000 cycles
Lignin/PAN (60/40) in DMF [168]	Stabilization: 250 °C, 1 °C/min, air Carbonization: 800 °C, 10 °C/min, 1h, N_2_ Air plasma treatment	N/A	SSA: 689 m^2^/g	6 M KOH	C_s_: 344.6 F/g at 1 A/g
Esterified lignin/PAN (50/50) in DMF [169]	Stablization: 200 °C, 0.2 °C/min, 1 h, air Carbonization:1000 °C, 5 °C/min, 30 min, N_2_ Activation: 600 °C, 1 h	KOH	SSA: 2313 m^2^/g Pore size: 1–3 nm	6 M KOH	C_s_: 320 F/g at 1 A/g
Lignin/PAN (50/50) in DMF [170]	Stabilization: 220 °C, 0.5 °C/min, 4 h, air Carbonization: 1000 °C, 4 °C/min, 4 h, N_2_ Activation: 800 °C, 1 h	KOH	SSA: 2042.86 m^2^/g Pore size: 1.8–6 nm	6 M KOH	C_s_: 428.9 F/g at 1 A/g
Lignin/PVA (75/25) in water [171]	Stabilization: 220 °C, air Carbonization: 800 °C, 1000 °C, 1200 °C, 1400 °C, N_2_	N/A	SSA: 797–1672 m^2^/g Pore size: 1.5–2.2 nm	PVA/H_3_PO_4_	C_s_: 60.4 F/g at 80 mA/g
Lignin/PVA (30/70, 50/50, and 70/30) in water [172]	Stabilization: 220 °C, air Carbonization: 1200 °C, 5 °C/min, 1 h, N_2_	N/A	SSA: 583 m^2^/g Total pore volume: 0.289 cm^3^/g	6 M KOH	C_s_: 64 F/g at 400 mA/g Capacitance retention: 90%, 6000 cycles
Lignin/PVA (90/10, 80/20, 75/25) in water [173]	Stabilization: 250 °C, 2 °C/min, 1 h, air Carbonization: 1000 °C, 5 °C/min, 1 h, He Activation: 800 °C, 130 °C/min, CO_2_/N_2_	CO_2_/N_2_	SSA: 1370–2170 m^2^/g Micropore volume: 0.36–0.53 cm^3^/g Mesopore volume: 0.09–0.365 cm^3^/g	Pyr_14_TFSI ionic liquid:PC:EC = 3:3:2	C_s_: 87 F/g at 10 mV/s Energy density: 38 Wh/kg at power density of 1666 W/kg Capacitance retention: 87%, 1000 cycles
Lignin/PVA (75/25) in water [174]	Stabilization: 250 °C, 4 °C/min, 2 h, air Carbonization: 600 °C, 4 °C/min, 1 h, N_2_ Activation: 900 °C	KOH	SSA: 1387–2005 m^2^/g Total pore volume: 0.60–0.71 cm^3^/g	0.5 M Na_2_SO_4_	C_s_: 205 F/g Capacitance retention: 83%, >1500 cycles
Lignin/PVA (~93/7) in water/accetic acid/MnO_2_ decoration [175]	Stabilization: 220 °C, 0.5 °C/min, 20 h, air Carbonization: 800, 900 and 1000 °C, 5 °C/min, 1 h, N_2_	N/A	SSA: 210–385 m^2^/g Total pore volume: 0.09–0.12 cm^3^/g	1M Na_2_SO_4_	C_s_: 131.28 F/g Energy density: 14.77 Wh/kg at power density of 135.01 W/kg at 0.3 A/g
Lignin/PVA (70/30) in water/MnO_2_ [176]	Stabilization: 220 °C, air Carbonization: 1200 °C, 5 °C/min, 1 h, N_2_	N/A	SSA: 583 m^2^/g	1.0 M LiPF_6_ in EC/DC/DMC (volume ratio 1:1:1)	C_s_: 83.3 F/g at 0.25 A/g Energy density 84.3 Wh/kg at power density of 5.72 kW/kg
Lignin/PVA (70/30) in water/surfactants (anionic, cationic, nonionic) [177]	Stabilization: stepwise, 105 °C, 5 °C/min, 1 h; 180 °C, 1 °C/min, 16 h; 220 °C, 0.5 °C/min, 6h, air Carbonization: 900 °C, 5 °C/min, 1 h, N_2_	N/A	N/A	6 M KOH	C_s_: 80.7 F/g at 0.1 A/g
Lignin/PEO (35/0.2) in DMF [178]	Stabilization: 250 °C, air Carbonization: 600 °C, N_2_ Activation: 250 °C, 20 °C/min; 1000 °C, 10 °C/min, 1 h, N_2_	KOH	SSA: 132–736 m^2^/g Total pore volume: 0.86–0.92 cm^3^/g	Electrode: KOH SC: KOH/PVA	C_s_ of electrode: 0.41 F/cm^2^ at 0.2 mA/cm^2^ C_s_ of device: 86.8 F/g at 1 A/g; capacitance retention: 60.5% at 10 A/g
Lignin/PEO (1:0.14) in NaOH/NaNO_3_ [179]	Carbonization/Activation: 800 °C, 2 h, 3 °C/min, 5% H_2_/N_2_	NaOH	SSA: 905–1249 m^2^/g Total pore volume: 0.38–0.52 cm^3^/g	6 M KOH	C_s_: 192 F/g at 0.1 A/g Energy density: 2245 μWh/cm^3^ Cyclic stability: 92.5%, 6000 cycles
Lignin/PEO (95/5) in DMF/ferric acetylacetonate (Fe(acac)_3_) [180]	Stabilization: 250 °C, 0.2 °C/min, 1 h, air Carbonization: 900 °C, 10 °C/min, 1 h, N_2_	N/A	N/A	1 M Na_2_SO_3_	C_s_: 72.1 F/g at 0.5 A/g Cyclic stability: 107%, 1000 cycles
Lignin/PEO (9/1) in NaOH [181]	Carbonization/Activation: 800 °C, 5 °C/min, 2 h	NaOH	SSA: 642 m^2^/g Total pore volume: 0.312 cm^3^/g	6 M KOH	C_s_: 180 F/g at 1 A/g
Lignin/PEO (9/1) in water/NaOH and KOH [182]	Carbonization/Activation: 850 °C, 10 °C/min, 0.5 h	NaOH and KOH	N/A	6 M KOH	C_s_: 344 F/g at 1 A/g Energy density: 8.1 Wh/kg Cyclic stability: 96%, 5000 cycles
Lignin/PEO (~9/1) in DMF for core; TEOS/PVP (60/40) in acetic acid/ethanol (2/15) for shell [183]	Carbonization: 900 °C, 5 °C/min, 2 h, N_2_	N/A	SSA: 870 m^2^/g	EMIMBF_4_	C_s_: 133 F/g at 1 A/g Energy density: 56.6 Wh/kg at power density of 114 W/kg
Lignin/PEO/PVP (57/3/12) in DMF/iron oxide [184]	Stabilization: 250 °C, 0.2 °C/min, 1 h, air Carbonization: 900 °C, 3 °C/min, 1 h, N_2_	N/A	SSA: 852–1463 m^2^/g Total pore volume: 0.3–0.57 cm^3^/g	6 M KOH	C_s_: 148 F/g at 0.5 A/g Energy density: 12.29 Wh/L Cyclic stability: 92.8%, 5000 cycles
Lignin/cellulose acetate (50/50) in acetone/DMF (2/1, *v*/*v*) [151]	Stabilization: 220 °C, 0.4 °C/min, 12 h, air Carbonization: 1400 °C, 4 °C/min, 2 h, N_2_	N/A	SSA: 306–1014 m^2^/g Total pore volume: 0.065–0.151 cm^3^/g Pore size: 1.6–3.1 nm	6 M KOH	C_s_: 175 F/g at 1 A/g Energy density: 6 Wh/kg at power density of 450 W/kg
Phosphatized lignin/cellulose acetate (50/50) in acetone/DMF (1/1, *v*/*v*) [152]	Stabilization: 220 °C, 2 °C/min, 2 h, air Carbonization: 800 °C, 5 °C/min, 1 h, N_2_	N/A	SSA: 829–2140 m^2^/g Total pore volume: 0.16–0.49 cm^3^/g	6 M KOH	C_s_: 363 F/g at 1 A/g Energy density: 31.2 Wh/kg at power density of 800 W/kg
Lignin/cellulose acetate (50/50) in acetone/DMAc (1/1, *w*/*w*)/H_3_PO_4_ [153]	Stabilization: 220 °C, 2 °C/min, 2 h, air Carbonization: 800 °C, 5 °C/min, 1 h, N_2_	N/A	SSA: 221–837 m^2^/g Total pore volume: 0.12-0.49 cm^3^/g	6 M KOH	C_s_: 347 F/g at 1 A/g Energy density: 31.5 Wh/kg at power density of 400 W/kg
Lignin/cellulose acetate (50/50) in acetone/DMF (2/1, *w*/*w*)/epichlorohydrin [185]	Stabilization: 220 °C, 0.4 °C/min, 12 h, air Carbonization: 600 °C, 4 °C/min, 2 h, N_2_	N/A	SSA: 553–1062 m^2^/g Total pore volume: 0.29–0.57 cm^3^/g	6 M KOH	C_s_: 320 F/g at 1 A/g Energy density: 30.2 Wh/kg at power density of 400 W/kg
Hardwood lignin/PEG (99/1, 95/5) in DMF/acetic acid (6/4)/hexamethylenetetramie crosslinker [186]	Stabilization: 250 °C, 0.5 and 2 °C/min, 1 h, air Carbonization: 900 °C, 3 °C/min, 1 h, N_2_ Activation: 900 °C, 10 °C/min, 1 h	H_2_O steam	SSA: 854–1509 m^2^/g Total pore volume: 0.44–1.07 cm^3^/g	EMIMBF_4_	C_s_: 227.3 F/g at 1 A/g Energy density: 91.5 Wh/kg at power density of 76.2 kW/kg
Lignin/protein (20/80, 50/50, 80/20) in acetic acid/DMF (90/10, *v*/*v*) [187]	Stabilization: stepwise, 200 °C, 1 °C/min; 250 °C, 0.5 °C/min, 3 h, air Carbonization: stepwise, 250 °C, 1 °C/min; 900 °C, 5 °C/min, 2 h, Ar/H_2_ Activation: 850 °C, 3 h	CO_2_	SSA: 561–1113 m^2^/g	6 M KOH	C_s_: 410 F/g at 1 A/g Cyclic stability: 95%, 3000 cycles
Lignin/PVP (1/2) in DMF/Mg(NO_3_)_2_·6H_2_O [188]	Pre-carbonization: stepwise, 150 °C, 1 °C/min, 24 h; 350 °C, 1 °C/min, 4 h, air Carbonization: 800 °C, 3 °C/min, 1 h, N_2_	N/A	SSA: 283–579 m^2^/g Total pore volume: 0.283–0.627 cm^3^/g	6 M KOH	C_s_: 248 F/g at 0.2 A/g Cyclic stability, 97%, 1000 cycles
Alkali lignin/PAN (3/1) in DMF as core; 9 wt.% SnCl_2_·2H_2_O/PVP in DMF as shell [189]	Stabilization: 200 °C, 300 °C or 400 °C, 2 h, air Carbonization: 800 °C	N/A	SSA: 554 m^2^/g	6 M KOH	C_s_: 229 F/g at 0.2 A/g Capacitence retention: 99%, 10,000 cycles Energy density: 7.2 Wh/kg at power density of 3.6 kW/kg
Lignin/PMMA (1/9, 3/7, 5/5, 7/3 and 9/1) in DMF as core; 9 wt.% SnCl_2_·2H_2_O/PVP in DMF as shell [190]	Stabilization: 300 °C, 1 °C/min, air Carbonization: 800 °C, 2 h, N_2_	N/A	SSA: 164–659 m^2^/g Total pore volume: 0.15–0.56 cm^3^/g	6 M KOH	C_s_: 406 F/g at 0.5 A/g Cyclic stability: 95%, 10,000 cycles

N/A: not available; PVA: poly(vinyl alcohol); PEO: polyethylene oxide; PAN: polacrylonitrile; PMMA: poly(methyl methacrylate); PEG: polyethylene glycol; PVP: poly(N-vinyl-2-pyrrolidone); DMF: dimethylformamide; THF: tetrahydrofuran; EC: ethylene carbonate; DC: diethyl carbonate; DMC: dimethyl carbonate; Pyr_14_TFSI: N-methyl pyrrolidinium bis(trifluoromethanesulfonyl)imide; PC: propylene carbonate; TEOS: tetraethyl orthosilicate; EMIMBF_4_: 1-ethyl-3-methylimidazolium tetrafluoroborate; DMAc: N,N-dimethylacetamide; SSA: specific surface area; C_s_: specific capacitance; SnCl_2_·2H_2_O: stannous chloride dihydrate; N_2_: nitrogen atmosphere; Ar: argon atmosphere; H_2_: hydrogen atmosphere; He: helium; CO_2_: carbon dioxide; KOH: potassium hydroxide; NaOH: sodium hydroxide; Na_2_SO_3_: sodium sulfite; H_3_PO_4_: phosphoric acid; Mg(NO_3_)_2_·6H_2_O: magnesium nitrate hexahydrate; MnO_2_: manganese dioxide; LiPF_6_: lithium Hexafluorophosphate; Na_2_SO_4_: sodium sulfate; MnCl_2_: manganese chloride; ZnO: zinc oxide; NaNO_3_: sodium nitrate; H_2_O: water; NiCo_2_O_4_: nickel cobaltite; Ni: nickel; Mn: manganese.

However, it is typically quite difficult to use lignin as an independent raw material to yield biomass-derived carbon fibers primarily due to its low molecular weight, 3D amorphous molecular structure and poor flexibility. Alternatively, lignin-based carbon fibers were fabricated by blending it with other polymers for good spinnability, thermal stability and excellent flexibility. The commonly used blending polymers include polyacrylonitrile (PAN), PVA, and polyethylene oxide (PEO), etc., which are summarized in Table 4.

One of the most common binder polymers for lignin-based CNFs is PAN. It has good spinnability, linear structure, high molecular weight, which contribute to good mechanical properties of the resulting fiber mats. Lignin/PAN-based CNFs [161,162,169,170] have been used as flexible supercapacitor electrodes since lignin is effective in increasing SSA, and lowering the average fiber diameter [161]. To further enhance the EC performance, metal compounds, including metal oxides (i.e., MnO_2_ [191], NiCo_2_O_4_ [163], zinc oxide (ZnO) [166]), metal sulfide [164], metal chloride (i.e., manganese chloride (MnCl_2_) [165]) were employed as additives in lignin/PAN CNFs electrodes to offer pseudocapacitance. Another effective way is to introduce heteroatom into lignin/PAN-based CNFs. With the addition of nitrogen/sulfur co-doped graphene [167] or air plasma treatment [168] of CNFs, the increased heteroatom content contributed to enhanced EC performance (344.6 F/g at 1 A/g [168]) due to faradaic reactions.

PVA is also frequently blended with lignin to yield electrospun CNFs with good spinnability for a flexible supercapacitor electrode. Electrospun CNFs from lignin/PVA with various mass ratios have been achieved with large SSA [171,172,173,174]. The decomposition of PVA in high temperature treatment for pore-generation was stressed [174]. It was found that at 75/25 (*w*/*w*) lignin/PVA, phase separation occurred in as-spun fibers. Later in the carbonization process, PVA with abundant oxygen atoms, served as a sacrificial material to create microporosity. Apart from the activation for high SSA and porosity [173,174], metal oxide such as MnO_2_ has also been used as an additive in lignin/PVA CNFs electrodes to tune the fiber mats’ morphology and provide extra pseudocapacitance [175,176]. Interestingly, twisted electrospun lignin/PVA CNFs-based yarns were developed to increase the mechanical and electrical properties [192]. The highest number of twists (78 turn/cm) efficiently decreased the yarn diameter down to 127 μm, and increased the electrical conductivity (21.9 S/cm), and mechanical properties (tensile strength of 0.5 GPa, Modulus of 26 GPa). However, the electrochemical capacitance decreased from 7.73 F/g of the mat to <1 F/g of the highly twisted yarn system. Further investigation of twisted yarn in smart textiles as energy storage will be of great interest since it is possible that yarns are strong enough to endure a textile manufacturing process.

Lignin/PEO was electrospun to yield interconnected fiber structures, which were beneficial for the electron transport and conductive property of the carbon networks. High SSA and porosity after carbonization and activation [178,179,181,182] contribute to the fast ion transfer. Moreover, iron oxide incorporated into electrospun lignin/PEO offered extra pseudocapacitance [180]. These film-based electrodes exhibited an excellent specific capacitance as shown in Table 4.

Other polymers such as poly(N-vinyl-2-pyrrolidone) (PVP) [184,188,189], poly(methyl methacrylate) (PMMA) [190], polyethylene glycol (PEG) [186,193], protein [187] and cellulose acetate [151,152,153,185] have been used as blending polymers to fabricate lignin-based electrospun CNFs as flexible electrodes, and the corresponding fabrication condition and EC performance is summarized in Table 4. These electrodes demonstrated high EC performance mainly due to high SSA and porous structure.

Lignin-based 2D film flexible electrode has also been fabricated for supercapacitor applications from lignin-derived porous carbon/reduced graphene oxide (rGO) film [194,195]. The optimal heat-treatment temperature was 250 °C. The assembled flexible supercapacitor achieved areal specific capacitance of 324.5 mF/cm^2^ at 0.2 mA/cm^2^, and 91.8% capacitance retention after 1000 charge/discharge cycles [195]. Furthermore, MnO_2_ has been deposited on lignin-derived porous carbon/rGO film [194]. The highly porous lignin-derived porous carbon promoted SSA and electrolyte ions transport. Also, rGO sheets interconnecting with the bio-based carbon provided a conductive bridge for ion transport. The deposited MnO_2_ nanoflakes interconnected with each other to form a porous structure with significantly increased SSA. Thus, the composite electrode had a good EC performance with maximum specific capacitance of 1136 mF/cm^2^ at a current density of 1 mA/cm^2^. The carbon electrode from molybdenum disulfide (MoS_2_)-decorated lignin/PAN film by laser direct writing had enhanced areal capacitances up to 16 mF/cm^2^ (2.2 F/cm^3^) and at 10 mV/s [196]. Chemically modified lignin with sulfonic groups was transformed into porous lignin-based carbon with large SSA (3149 m^2^/g) after carbonization and chemical activation [197]. The heteroatoms and the graphitic structure had a profound impact on the EC performance. Thus, the flexible symmetric supercapacitor assembled by this electrode demonstrated capacitance of ~140 F/g at 0.5 A/g, high energy density of 5.41 Wh/kg at power density of 0.5 kW/kg.

Lignin-derived carbon aerogels were obtained by ultrafast freezing of lignin/KOH solution droplets, followed by freeze-drying and in situ activation during carbonization at 900 °C [198]. The resulting carbon aerogels had hierarchically porous structure (corresponding to a high SSA of 1681.6 m^2^/g) that facilitated ion diffusion and charge transport. Moreover, the abundant oxygenated groups provided additional pseudocapacitance that boosted the EC performance of supercapacitor electrodes: a high specific capacitance of 189 F/g at 1 A/g, a high energy density of 26.25 Wh/kg at a power density of 1000 W/kg, and a capacitance retention of 97.4% after 10,000 cycles.

**Figure 11:**
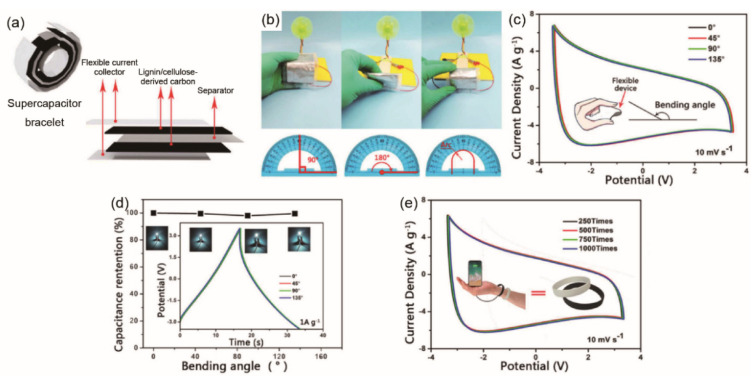
The EC performance of cellulose acetate-based flexible SC at different bending angles. (**a**) Schematic illustration of the flexible supercapacitor. (**b**) The small fan powered by the flexible supercapacitor at different bending angles. (**c**) CV curves under 10 mV/s. The inset depicts a bending angle. (**d**) Capacitance retentions and the corresponding bulb brightness. The inset shows the galvanostatic charge/discharge (GCD) curves at 1 A/g. (**e**) CV cycle test at 10 mV/s. The inset shows a supercapacitor wristband. (**a**–**e**) Reproduced with permission from ref. [199] Copyright 2019 John Wiley and Sons.

Three-dimensional (3D) lignin/cellulose-derived carbon aerogel electrode was pressed into film to fabricate symmetrical flexible supercapacitors for the application of wearable and portable energy storage devices (Figure 11) [199]. The devices revealed capacitance behavior and a stable power delivery at different bending angles (Figure 11c,d): a superior energy density of 102 Wh/kg at power density of 175 W/kg, and good capacitance retention (>99%). Kraft and soda lignins with cellulose nanofibers were converted to carbon aerogel. It was found that Kraft lignin-based carbon aerogel had better specific capacitance of 163 F/g and energy density of 5.67 Wh/kg at a power density of 50 W/kg when assembled into a two-electrode symmetrical supercapacitor due to higher SSA and hierarchically porous structure [200].

In summary, lignin-based carbon materials in fiber, nanofiber membrane and carbon aerogel configuration for high-performance flexible SC electrodes are potential high-value products for smart textiles.

### 5.3. Flexible Supercapcitors from Raw Lignocellulose-Derived Carbon

Flexible supercapacitors assembled from raw lignocellulose-derived carbon electrodes have indicated a facile way to fabricate low-cost wearable electronics for smart textiles. A large number of research articles regarding raw lignocellulose-derived AC electrodes with considerable EC performance have been reported [24,201]. Thus, it is possible to convert lignocellulosic raw materials into carbon electrodes of flexible supercapacitor with intrinsic porous structure; however, only a few works in the literature have focused on this aspect. Table 5 concisely summarizes the fabrication and EC performance of raw lignocellulose-derived carbon electrode for flexible supercapacitor applications.

One-dimensional (1D) fiber-shaped continuous carbon fiber electrode derived from raw lignocellulose stock has not been extensively investigated. Wet-spun wood lignocellulose nanofibrils/cellulose (cellulose loading of 0–33 wt.%) fibers were carbonized at 900 °C to achieve good electrical conductivity (66 S/cm), and excellent mechanical performance (~15 cN/tex) [202]. The specific capacitance of fiber-shaped supercapacitors was not very high (25 F/cm^3^), which was possibly due to low SSA (12–46 m^2^/g). However, they demonstrated a long-term electrochemical stability (>93% of the initial capacitance after 10,000 cycles).

AC derived from straw possessed nitrogen atoms and high SSA up to 1923 m^2^/g [203], which were beneficial for better EC performance. The resulting AC/GO film had a dense structure of 1.23 g/cm^3^ and good conductivity. The assembled flexible solid-state supercapacitor device had a volumetric capacitance of 326 F/cm^3^ at 0.5 A/g. Cane stalk [204] or sugarcane bagasse [205], porous lettuce slice [206], corncob sponge [207], soybean pod [208], kenaf stem [209], jute [210,211], fruit shell [212], and cotton [213] have also been directly converted to carbon film electrodes for flexible supercapacitors, which are summarized in Table 5. They all demonstrated good pore characteristics, which correspond to the excellent EC performance of flexible supercapacitors.

**Table 5:** Literature summary of flexible supercapacitors based on raw lignocellulose-derived carbon.

Raw Lignocellulose-Derived Carbon/Other Material (Mass Ratio) and Reference	Thermal Treatment	Activation Agent	Porosity Characteristics	Electrotyle	Electrochemical Performance
Straw-derived AC/GO (3/1) [203]	AC: pre-carbonization at 500 °C, 2 h, N_2_; activation at 700 °C, 2 h, N_2_ Composite: microwave, 180 °C, 1 h	KOH	SSA of AC: 1923 m^2^/g SSA of composites: 533 m^2^/g	1 M H_2_SO_4_	C_s_ of electrode: 775 F/cm^3^ at 0.5 A/g C_s_ of device: 326 F/cm^3^ at 0.5 A/g Volumetric and gravimetric energy densities: 9.7 Wh/L and 7.9 Wh/kg
Sugarcane bagasse-derived AC [205]	Carbonization/Activation: 800 °C, 10 °C/min, 1 h	KOH	SSA of AC: 1437–1534 m^2^/g	6 M KOH	C_s_: 185.5 F/g at 0.5 A/g; 150.7 F/g at 20 A/g Energy density: 1.02 Wh/kg at power density of 6120 Wh/kg Cycling performance: 96%, 10,000 cycles
Porous lettice-derived carbon [206]	Pre-carbonization: 260 °C, 5 °C/min, 6 h Carbonization: 800 °C, 900 °C, and 1000 °C, 5 °C/min, 2 h, N_2_	N/A	SSA: 683–1973 m^2^/g	6 M KOH	C_s_: 213.4 F/g at 0.2 A/g Cyclic performance: 96.9%, 100,000 cycles Energy density: 7.41 Wh/kg at power density of 50.4 W/kg
Waste cotton derived AC/carbon black [213]	Carbonization: 800 °C, 1 h, N_2_ Activation: 950 °C, 1 h, N_2_	KOH	SSA: 2350 m^2^/g Pore volume: 1.3 cm^3^/g	0.6 M NaCl	C_s_: 172 F/g at 1 A/g Cyclic stability: 99.9%, 10,000 cycles
Wet-spun lignocellulose/cellulose carbon fibers [202]	Carbonization: 900 °C, 5 °C/min, 1 h, N_2_	N/A	SSA: 12–46 m^2^/g Total pore volume: 0.0033–0.0094 cm^3^/g Pore size: 2.7–4.7 nm	PVA/H_2_SO_4_	C_s_: 25 F/cm^3^ at 10 mV/s Cyclic stability: >93%, 10,000 cycles Energy density: 0.25 mWh/cm at power density of 65.1 mW/cm
Cane stalk-derived porous carbon [204]	Carbonization: 300 °C, 5 °C/min, 1 h; 600 °C, 2 h, N_2_ Activation: 300°C, 5 °C/min, 1 h; 800 °C, 2 h	KOH, Fe(NO_3_)_3_·9H_2_O	SSA: 1902–1924 m^2^/g Total pore volume: 1.22–1.66 cm^3^/g	6 M KOH	C_s_: 514.14 F/g at 0.3 A/g; 372.57 F/g at 100 A/g Rate capability: 82.34% from 0.3 A/g to 100 A/g Cyclic stability: 101.51%, 5000 cycles
Nitrogen-sulfur co-doped corncob sponge-derived AC [207]	Carbonization/Activation: 400 °C, 2 h; 850 °C, 5 °C/min, 1 h, N_2_	KOH	SSA: 1540–1909 m^2^/g Total pore volume: 0.801–0.852 cm^3^/g	6 M KOH PVA/KOH	C_s_: 404 F/g at 0.1 A/g; 253 F/g at 10 A/g Energy density: 30 Wh/kg at 8 kW/kg Capacitence retention: 99%, 10,000 cycles at 3 A/g
Nitrogen-rich soybean pod-derived AC [208]	Carbonization: 500 °C, 2 h, N_2_ Activation: 800 °C, 2 h, N_2_	NaOH	SSA: 962–2612 m^2^/g Total pore volume: 0.613–0.838 cm^3^/g	1 M Na_2_SO_4_	C_s_ of electrode: 352.6 F/g at 0.5 A/g Flexible SC C_s_: 4.70 F/cm^2^ at 10 mA/cm^2^; capacitance retention: 93%, 10,000 cycles
Kenaf stem-derived AC [209]	Carbonization/Activation: 900 °C, 2 h	NiCl_2_	SSA: 1064–1480 m^2^/g Pore size: 3.48–9.96 nm	1 M H_2_SO_4_	C_s_: 327 F/g at 2 mV/s Cyclic performance: 95.6%, 5000 cycles Rate performance: 84.7% from 2 mV/s to 100 mV/s
Jute sticks-derived AC [210]	Precarbonization: 450 °C, Ar Carbonization/Activation: 800 °C, 900 °C, 1000 °C, and 1100 °C, 5 °C/min, 1 h, Ar	KOH	SSA: 949–2393 m^2^/g Total pore volume: 0.69–1.62 cm^3^/g	6 M KOH, 1 M NEt_4_BF_4_ in acetonitrile solution	C_s_: 282 F/g at 0.5 A/g Energy density: 20.6 Wh/kg at power density of 33.6 kW/kg
Jute fibers-derived AC [211]	Carbonization/Activation: 800 °C, 3 °C/min, 1 h, Ar	KOH	SSA: 1796 m^2^/g	3 M KOH	C_s_: 408 F/g at 1 mV/s Cyclic stability: 100%, 5000 cycles
Nitrogen-doped cotton-derived carbon cloth [214]	Carbonization: 1000 °C, 40 °C/min, 15 to 45 min, NH_3_	N/A	SSA: 468–2116 m^2^/g Total pore volume: 0.201–1.561 cm^3^/g	1 M TEABF_4_ in acetonitrile	C_s_: 215.9 F/g at 1 A/g Rate capability: 89% from 1 A/g to 200 A/g Cycling stability: 98%, 20,000 cycles
Nitrogen- and oxygen-doped porous carbon derived from cotton [215]	Activation: 800 °C, 5 °C/min, 2 h, N_2_ Oxidation: 300 °C, 350 °C and 400 °C, 5 h, air	DAP	SSA: 268–1022 m^2^/g Total pore volume: 0.228–0.534 cm^3^/g	6 M KOH	C_s_: 292 F/g at 0.5 A/g Capacitance retention: 87%, 5000 cycles at 5 A/g Energy density: 18 Wh/kg at 250 W/kg
AC derived from fruit shell [212]	Carbonization/Activation: 700 °C, 4 h, Ar	KOH	SSA: 673–1040 m^2^/g Total pore volume: 0.36–0.61cm^3^/g	PVA/H_2_SO_4_ with Na_2_MoO_4_	C_s_: 648 F/g at 1.56 A/g Energy density: 14.4 Wh/kg at 625 W/kg Capacitance retention: 93%, 3000 cycles

AC: activated carbon; GO: graphene oxide; NEt_4_BF_4_: tetraethylammoniumtetrafluoroborate; TEABF_4_: tetraethylammonium tetrafluoroborate; DAP: diammonium hydrogen phosphate; PVA: poly(vinyl alcohol); KOH: potassium hydroxide; H_2_SO_4_: sulfuric acid; NaCl: sodium chloride; Na_2_SO_4_: sodium sulfate; Na_2_MoO_4_: sodium molybdate; N_2_: nitrogen atmosphere; NH_3_: ammonia; Ar: argon atmosphere; NiCl_2_: nickel chloride; Fe(NO_3_)_3_·9H_2_O: ferric nitrate nonahydrate SSA: specific surface area; C_s_: specific capacitance.

Moreover, the surface chemistry of heteroatom-doping has been applied to enhance the EC performance of raw lignocellulose-derived flexible supercapacitor carbon electrodes. For example, nitrogen-doped AC cloth was achieved by ammonia treatment of commercial cotton fabrics at 1000 °C [214]. The resulting flexible supercapacitors assembled by this hierarchically porous carbon cloth had a high specific capacitance (up to 215.9 F/g at 1 A/g), high rate capability (89% from 1 A/g to 200 A/g), and excellent cycling stability (98% capacitance retention over 20,000 cycles). Oxygen/nitrogen-doped porous carbon based on cotton were prepared by the immersion of cotton in diammonium hydrogen phosphate (DAP), subsequent activation at 800 °C and oxidation in 300–400 °C [215]. High SSA (1022 m^2^/g) and relatively high pore volume (0.53 cm^3^/g) were obtained, and optimal electrodes oxidized at 350 °C had high specific capacitance of 292 F/g at a current density of 0.5 A/g in a three-electrode system. The symmetrical supercapacitor had a high stability with 87% of capacitance retained after 5000 cycles at 5 A/g, as well as a high volumetric energy density (18 Wh/kg at 250 W/kg).

In summary, flexible carbon electrodes from raw lignocellulosic biomass with high SSA, and hierarchically porous structures are promising low-cost bio-based electrodes for flexible supercapacitors. Carbonization, activation and heteroatom-doping greatly influence the surface chemistry and microstructures of electrodes, and enhance the EC performance of the resulting flexible supercapacitors.

## 6. Supercapacitors from Lignocellulose-Based Graphite

A high level of graphitization of lignocellulosic biomass-derived carbon is preferred to achieve high electrical conductivity, low internal resistance of electrode materials and fast charge transfer, and thereby enhanced EC performance. Lignocellulosic graphite can be obtained by high temperature treatment [216], which increases sp^2^ carbon atoms in the heating process. However, excessively high temperatures may cause the collapse of pores, which reduces SSA, pore volume, and surface functional groups, negatively affecting the EC performance of the electrodes [28].

The preferred method to obtain highly graphitized carbon from lignocellulose is catalytic graphitization during activation with different catalysts such as ferric chloride (FeCl_3_) [217,218], ferric nitrate (Fe(NO_3_)_3_) [219,220], potassium ferrate (K_2_FeO_4_) [221,222], potassium carbonate (K_2_CO_3_) [223], and nickel chloride (NiCl_2_) [224,225], etc. Table 6 summarizes some examples of supercapacitors based on lignocellulose-derived graphitic carbon including the raw material, thermal treatment, catalyst, structural characteristics and EC performance, etc. For instance, graphitic bamboo char was achieved at 800 °C, with K_2_FeO_4_ acting both as activator and graphitization catalyst. The resulting electrode had a porous structure with SSA of 1732 m^2^/g, and good electrical conductivity of 4.7 S/cm. These characteristics significantly contributed to the enhanced EC performance [221]. Similarly, graphite from coconut shells was obtained with K_2_CO_3_ acting as activator/graphitization catalyst simultaneously at 900 °C [223]. The resulting graphene-like sheets had high SSA (1506 m^2^/g), and excellent electrical conductivity (32.14 S/cm) due to high degree of graphitization with an ordered structure. Potassium in the catalyst catalyzes the crystallization of amorphous carbon, and forms charge transfer complexes with aromatic hydrocarbons to achieve catalytic graphitization with increased sp^2^ hybridized valences.

Graphitic carbons with a hierarchical porous structure often have elevated ion/electrons conductivity, and thereby excellent EC performance. For example, hierarchically porous graphitic carbon derived from rice straw with interconnected pores had significant SSA of 3333 m^2^/g, abundant micro-/mesopores contributing to high specific capacitance (400 F/g), as well as good cycling stability [226]. Porous graphitic carbon from willow catkins was obtained at 900 °C with potassium hexacyanoferrate (II) (K_4_Fe(CN)_6_) as activator/graphitization catalyst. The resulting material had a high graphitization degree (*I_D_/I_G_* ~0.82) and SSA (1067 m^2^/g), which were much better than those of willow catkins-derived carbon without K_4_Fe(CN)_6_ (*I_D_/I_G_*~0.87 and 31.54 m^2^/g, respectively) [227].

To further increase the EC performance of lignocellulose-derived graphitic carbon, heteroatom-doping can be used. For instance, one-step carbonization and graphitization of lotus leaf by FeCl_3_, together with heteroatom-doping resulted in supercapacitors with high specific capacitance of 385 F/g at 0.5 A/g [218]. This was mainly due to the high SSA, high level of graphitization (*I_D_/I_G_*~1.04), good electrical conductivity (8.7 S/cm) and extra psuedocapacitance by heteroatom-doping. Similarly, nitrogen-doped porous graphite carbon from peach gum [225] obtained at 700 °C by NiCl_2_ had a high specific capacitance of 426 F/g at 0.5 A/g.

In summary, factors including graphitization degree, SSA, pore volume, and surface chemistry should be carefully considered to obtain supercapacitors from lignocellulose-derived graphite with optimized EC performance.

**Table 6:** Examples of supercapacitors based on lignocellulose-derived graphite.

Raw Material and Reference	Thermal Treatment	Graphitization Catalyst	Pore Characteristics	Graphitization Degree	Electrical Properties	Electrolyte	Electrochemical Performance
Beech wood-derived graphite [217]	500 °C, 1 °C/min; 1000 °C, 5 °C/min, 0.5 h, N_2_	FeCl_3_	SSA: 370 m^2^/g Total pore volume: 0.24 cm^3^/g	N/A	Electrical resistivity: 2.2 × 10^−4^ Ω·m	1 M KOH	C_s_: 133 F/g at 20 mA/g; 35 F/g at 1 A/g
Nitrogen, Sulfur-doped lotus leaf-derived graphitic carbon [218]	750 °C, 5 °C/min, 2 h, Ar	FeCl_3_	SSA: 976 m^2^/g Total pore volume: 0.55 cm^3^/g	*I_D_/I_G_*: ~1.04	Electrical conductivity: 8.7 S/cm	6 M KOH	C_s_: 385 F/g at 0.5 A/g Energy density: 29.5 Wh/kg at power density of 545.6 W/kg Cycling stability: 95.3%, 20,000 cycles at 5 A/g)
Porous graphitic carbon from chopstick sawdust [220]	850 °C, 2 h, N_2_	Fe(NO_3_)_3_ and K_2_C_2_O_4_	SSA: 2187 m^2^/g Total pore volume: 2.38 cm^3^/g	*I_D_/I_G_*: ~0.95	N/A	6 M KOH	C_s_: 231.1 F/g at 0.2 A/g Cycling stability: 96.6%, 10,000 cycles
Bamboo char-derived porous graphitic carbon [221]	800 °C, 5 °C/min, 2 h, Ar	K_2_FeO_4_	SSA: 1732 m^2^/g Total pore volume: 0.97 cm^3^/g Micropore volume: 0.80 cm^3^/g	*I_D_/I_G_*: ~0.62	Electrical conductivity: 4.7 S/cm	KOH/PVA	C_s_: 222.0 F/g at 0.5 A/g Energy density: 6.68 Wh/kg at power density of 100.2 W/kg; 3.33 Wh/kg at 10 kW/kg
Fallen leaves of phoenix tree [222]	650, 800 and 900 °C, 2 h, Ar	K_2_FeO_4_	SSA: 2208 m^2^/g Total pore volume: 0.97 cm^3^/g Micropore: >71.8%	*I_D_/I_G_*: ~0.93	Electrical conductivity: 2.38 S/cm	6 M KOH; 1M H_2_SO_4_	C_s_: 254 F/g (KOH), 273 F/g (H_2_SO_4_) at 0.5 A/g
Coconut shell biochar-derived graphite [223]	900 °C, 10 °C/min, 2 h, N_2_	K_2_CO_3_	SSA: 1506 m^2^/g Pore size: 0.5–4.5 nm	*I_D_/I_G_*: ~0.15	Electrical conductivity: 32.14 S/cm	TEMABF_4_/PC	C_s_: 91.15 F/g at 0.2 A/g Cycling stability: 91%, 5000 cycles at 0.1 A/g
Nitrogen-doped porous graphite carbon from peach gum [225]	700 °C, 2 h, N_2_	NiCl_2_	SSA: 1161 m^2^/g Total pore volume: 1.67cm^3^/g	*I_D_/I_G_*: ~0.93	Electrical conductivity: 11.43 S/cm	6 M KOH	C_s_: 426 F/g at 0.5 A/g Cyclic stability: 97.09%,10,000 cycles at 20 A/g
Willow catkins-derived porous graphitc carbon [227]	900 °C, 20 °C/min, 2 h, N_2_	K_4_Fe(CN)_6_	SSA: 1067 m^2^/g Total pore volume: 0.75 cm^3^/g	*I_D_/I_G_*: ~0.82	N/A	1 M Na_2_SO_4_	C_s_: 550.8 F/g at 2 A/g Rate capability: 61.8% at 50 A/g Cycling stability: 89.6%, 5000 cycles at 10 A/g
Porous graphitic carbon derived from rice straw [226]	700 °C, 1 h, 10 °C/min, N_2_	Ni(NO_3_)_2_	SSA: 3333 m^2^/g Total pore volume: 2.16 cm^3^/g	*I_D_/I_G_*: ~0.84	N/A	6 M KOH	C_s_: 400 F/g at 0.1 A/g Cyclic stability: 93.6%,10,000 cycles

N/A: not available; *I_D_/I_G_*: intensity ratios of the D (disordered carbon) band and G (graphitic carbon) band in Raman spectrum; TEMABF_4_: triethylmethylammonium tetrafluoroborate; PC: propylene carbonate; SSA: specific surface area; C_s_: specific capacitance; FeCl_3_: ferric chloride; Fe(NO_3_)_3_: ferric nitrate; K_2_C_2_O_4_: potassium oxalate; K_2_FeO_4_: potassium ferrate; K_2_CO_3_: potassium carbonate; NiCl_2_: nickel chloride; Ni(NO_3_)_2_: nickel nitrate; K_4_Fe(CN)_6_: potassium hexacyanoferrate (II); KOH: potassium hydroxide; PVA: poly (vinyl alcohol); Na_2_SO_4_: sodium sulfate; H_2_SO_4_: sulfuric acid; N_2_: nitrogen atmosphere; Ar: argon atmosphere.

## 7. Future Direction

Due to the inherent structural properties of lignocellulose-based materials, it is possible to utilize a simple, scalable heat-treatment process to obtain bio-based carbon/graphite electrodes for flexible/wearable supercapacitors with different configurations. Even though lignocellulose is quite cost effective, a flexible fiber/yarn-shaped carbon electrode with high SSA, pore volume and sufficient mechanical performance for textile manufacturing process has rarely been demonstrated. Future advances towards lignocellulose-derived carbon electrode for flexible supercapacitors are listed below.

1. A green manufacturing process and product safety of flexible supercapacitors should be addressed. As mentioned, physical and chemical activations of lignocellulose-derived carbon are common methods for the preparation of porous carbon electrodes with preferably microporous structures. However, energy conservation and hazards should be considered. Chemical activation reagent, such as KOH, reacts with the carbon matrix at high temperatures and generates a large number of volatile gases, as well as metallic potassium. This may cause hazard corrosion issues. Also, due to the corrosive nature of most of the electrolytes (i.e., H_2_SO_4_, KOH) used in flexible supercapacitors, strict packaging is required before the final application of flexible supercapacitors in smart textiles. This may cause discomfort, laundering difficulties and electrolyte leaking problems in smart textile applications. Thus, greener activation strategies and environmentally friendly electrolyte should be developed to promote scalable production of porous biomass-derived carbon for supercapacitor applications.
2. Flexible lignocellulose-derived carbon electrode is mainly achieved by film, electrospun mats, aerogel or existing cloth. It is still rare that inherently functional, continuous lignocellulose-derived carbon fibers/yarn are employed in supercapacitor applications possibly due to the difficulty of fiber spinning/yarn formation. Typically, continuous fibers are achieved by textile fiber spinning techniques, which prefer dense fiber structure with no pores. This contradicts the need for the formation of hierarchically porous electrode with high EC performance. Thus, fiber spinning/yarn formation processes or surface chemistry should be more systematically investigated to form continuous fiber/yarn with sufficient pores and mechanical strength.
3. The enhancement of the mechanical performance of flexible supercapacitors is still an issue to be addressed. The vulnerability to withstand an intense textile manufacturing process such as weaving or knitting is still an obstacle that hinders the wide application of flexible supercapacitors in practical smart textiles. To solve this problem, the mechanical performance of fiber-, yarn- or fabric-based lignocellulose-derived carbon/graphite electrodes should be significantly investigated and enhanced. A comprehensive understanding of the structure–property relationships of lignocellulose, lignocellulose-derived carbon/graphite and the resulting fiber-, yarn- or fabric-based flexible supercapacitors is required. In particular, the revelation of the relationships of a material’s molecular arrangement, micro- to macro-structure and mechanical properties, will be beneficial for an expanded application of biopolymers in flexible energy storage systems.

With the development of a green manufacturing process, along with progress of inherently functional continuous fiber/yarn formation, and enhancement of the mechanical performance of lignocellulosic carbon/graphite electrode, it is very promising that lignocellulosic stock will be a renewable resource for the manufacture of low-cost flexible supercapacitor carbon electrodes for smart textile applications.

## Figures and Tables

**Table 1:** Comparison of specific surface area (SSA) and pore volume for unactivated and activated carbon materials.

Activation		Material and Reference	SSA (m^2^/g)		Pore Volume (cm^3^/g)	
Method	Activation Condition		Before Activation	After Activation	Before Activation	After Activation
Chemical activation	NaOH and KOH activation agents, 850 °C, 0.5 h, N_2_	Lignin-based carbon fiber [74]	13	1444	0.015	0.91
	80% H_3_PO_4_ activation agent, 250 °C, 2 h, air	Carbonate-free oil shale [75]	13	587	N/A	N/A
Physical activation	H_2_O steam activation agent, 800 °C, 90 min, N_2_	Epoxy resin-based carbons [76]	221	883	0.137	0.49
	H_2_O steam activation agent, 700–900 °C, 1 h, N_2_	Sludge-based adsorbents [77]	14	422	0.04	0.50

N/A: not available; KOH: potassium hydroxide; NaOH: sodium hydroxide; H_3_PO_4_: phosphoric acid; N_2_: nitrogen atmosphere; H_2_O: water.

## Data Availability

No new data were created or analyzed in this study. Data sharing is not applicable to this article.
